# Evolutionarily conserved hydrophobicity and sterics in TM3/TM4 balance Orai1 pore opening

**DOI:** 10.1002/pro.70684

**Published:** 2026-07-12

**Authors:** Maximilian Fröhlich, Tamara Radišković, Valentina Hopl, Magdalena Prantl, Hadil Najjar, Selina Harant, Diana Thallinger, Muhammad Shehzad Anwar, Ferdinand Horvath, Julia Söllner, Yuliia Nazarenko, Matthias Sallinger, Nora Müller, Adéla Tiffner, Sarah Weiß, Herwig Grabmayr, Heinrich Krobath, Isabella Derler

**Affiliations:** ^1^ JKU Life Science Center, Institute of Biophysics, Johannes Kepler University Linz Linz Austria; ^2^ Institute of Theoretical Physics, Johannes Kepler University Linz Linz Austria; ^3^ Present address: Institute for Physiology and Pharmacology, Johannes Kepler University Linz Krankenhausstraße 5 Linz 4020 Austria; ^4^ Present address: Institute of Science and Technology Austria Am Campus 1 Klosterneuburg 3400 Austria

**Keywords:** CRAC channel, hydrophobicity, Orai1, pore opening, sterics

## Abstract

The calcium (Ca^2+^) channel Orai1—the pore‐forming subunit of the Ca^2+^ release‐activated Ca^2+^ channel—is opened by its physiological activator, the endoplasmic reticulum (ER)‐resident Ca^2+^ sensor stromal interaction molecule 1 (STIM1) upon ER Ca^2+^ store‐depletion. STIM1 primarily engages the Orai1 C‐terminus, relaying an activation signal from the channel periphery via all transmembrane (TM) domains to the Ca^2+^‐conducting pore. We previously demonstrated that STIM1 coupling elicits conformational changes in the Orai1 TM3/TM4‐interface inducing pore opening. Here, we reveal that a subtle balance of steric and hydrophobic interactions around V181 (TM3), F250 and F253 (TM4) is required for proper physiological function. We show that side chain volume and hydrophobicity in this region jointly determine not only the stability of the channel complex in the closed state, but also the extent of Ca^2+^ influx. In physiological settings, this fine‐tuning around V181 permits sufficient activation, while preventing excessively large Ca^2+^ current.

## 1 INTRODUCTION

Calcium (Ca^2+^) is indispensable for all living organisms, acting as a cytosolic messenger that orchestrates a wide range of processes, including cell differentiation, proliferation, transcriptional activation, and apoptosis (Berridge et al., 2000; Berridge et al., 2003). In the human body, Ca^2+^ deficiency can manifest as neuromuscular irritability, cardiomyopathy, and various ectodermal changes such as dermatitis or hair loss (Schafer & Shoback, 2000; Schafer & Shoback, 2013). Conversely, excessive Ca^2+^ levels are linked to depression, muscle weakness, and autoimmune disorders, such as rheumatoid arthritis (Feske, 2019; Lacruz & Feske, 2015; Oelzner et al., 2006; Lu et al., 2019). Given these diverse effects, intracellular Ca^2+^ levels must be tightly regulated to maintain cell function and organismal health. This precise control of Ca^2+^ homeostasis is maintained by a series of ion channels (Wang et al., 2021), among which the Ca^2+^ release‐activated Ca^2+^ (CRAC) channel plays a prominent role (Derler et al., 2016; Fahrner et al., 2013; Najjar et al., 2026; Prakriya & Lewis, 2015; Tiffner et al., 2021a).

The CRAC channel consists of two interacting proteins: the Stromal Interaction Molecule (STIM), which resides in the membrane of the endoplasmic reticulum (ER) sensing intraluminal Ca^2+^ levels, and Orai, which forms a highly Ca^2+^ selective pore in the plasma membrane (PM) (Liou et al., 2005; Najjar et al., 2026; Prakriya et al., 2006; Sallinger et al., 2024). In humans, two isoforms of STIM (STIM1 and STIM2; Roos et al., 2005; Zhang et al., 2005) and three isoforms of Orai (Orai1–3; Vig et al., 2006a; Feske et al., 2006) have been identified as components of the physiological CRAC channel (Feske et al., 2006). In this work, we focus on the human STIM1 and Orai1 (hSTIM1 and hOrai1), hereafter referred to as STIM1 and Orai1.

CRAC channels are activated in response to receptor stimulation at the membrane. This initiates the binding of a second messenger molecule inositol 1,4,5 trisphosphate (IP_3_) to its receptor on the ER membrane, causing the Ca^2+^ release from the ER, the largest cellular Ca^2+^ store (Kuno & Gardner, 1987; Lewis, 2001; Mikoshiba, 2000; Vig et al., 2006a; Zhang et al., 2006). In response to store‐depletion, STIM1 undergoes oligomerization, which causes a conformational extension of STIM1. This allows STIM1 to translocate to ER‐PM junctions, where it couples to Orai1 to activate Ca^2+^ influx (Luik et al., 2008; Navarro‐Borelly et al., 2008).

From a structural perspective, the Orai1 channel is a hexamer, as revealed by the closed state X‐ray crystallography structure of *Drosophila melanogaster* Orai (dOrai), which shares 73% sequence similarity to Orai1 (Hou et al., 2012). Consistently, two open state mutants, dOrai H206A (corresponding to Orai1 H134) and dOrai P288L (corresponding to Orai1 P245L), which have been resolved using both X‐ray crystallography (Hou et al., 2018; Liu et al., 2019) (PDB: 6BBF and PDB: 6AKI, respectively) and cryo‐electron microscopy (PDB: 7HR5 for dOrai H206A) (Hou et al., 2020) exhibit a hexameric symmetry. Each subunit comprises four transmembrane domains (TM1–4) connected via one intracellular and two extracellular loops and flanked by the cytosolic N‐ and C‐terminus (Feske et al., 2006). TM1 directly lines the central pore and is surrounded by TM2 and TM3, while TM4 constitutes the peripheral part (Feske et al., 2006; Hou et al., 2018). The Orai1 C terminus serves as the main STIM1‐binding site (Muik et al., 2008; Park et al., 2009), while the N‐terminus and the cytosolic loop (loop2) also contribute to STIM1‐mediated gating (Butorac et al., 2019; Derler et al., 2018; Fahrner et al., 2018; McNally et al., 2013). Concerning the latter cytosolic regions, definitive mechanisms and contact sites mediating their interplay with STIM1 remain to be determined, in part due to the lack of high‐resolution structures of full‐length Orai1 complexed with STIM1.

Comparison of the closed and open state structures together with functional studies suggests that STIM1 binding to the Orai1 C‐terminus triggers a wave of coordinated motions propagated via multiple gating checkpoints across all four TM domains to induce pore opening (Tiffner et al., 2021b). This global conformational change of the entire Orai1 channel leads, on the one hand, to structural changes in the center of the complex, involving the rotation of pore‐lining TM1 (Yeung et al., 2018) around the hydrophobic cavity and a widening of its cytosolic, basic region (Frischauf et al., 2017; Hou et al., 2012; Hou et al., 2018; Liu et al., 2012).

On the other hand, the propagation of the activation signal from the C‐terminal STIM1 coupling site to the pore drives structural rearrangements in the individual non‐pore‐lining TM domains (Tiffner et al., 2021b; Yeung et al., 2018). Key gating sites along the peripheral TM4‐C‐terminal segment are (i) P245 in TM4, linked to the Stormorken‐like syndrome and tubular aggregate myopathy (Nesin et al., 2014), which forms a shallow bend in the middle of TM4 and (ii) the nexus region connecting TM4 with the C‐terminus via a sharp bend (Zhou et al., 2016). It is supposed that STIM1 binding leads to the straightening of the TM4‐C‐terminus segment (Hou et al., 2018; Palty & Isacoff, 2015), whereas the physiologically relevant extent of these structural rearrangements remains still unknown.

Another crucial gating checkpoint is located in close proximity to P245 and is V181 in TM3. Previous reports show that substitutions at this position can profoundly impact Orai1's functionality (Hopl et al., 2024; Tiffner et al., 2020; Tiffner et al., 2021b; Tiffner et al., 2021c). Mutants such as Orai1 V181S/A exhibit weak constitutive activity, while introduction of some charged residues at this position leads to robust gain of function (GoF) likely due to attraction of water molecules. This water‐mediated effect was proposed to cause dilation of the peripheral TM interfaces, and, as a result, an opening of the pore (Hopl et al., 2024).

To overcome the limitation of static insights into activation states upon conventional mutagenesis and to gain more dynamic insights into the Orai1 pore opening mechanism, we recently utilized the genetic code expansion (GCE) technique together with light‐sensitive unnatural amino acids (UAAs) (Coin, 2018). The site‐specific UAA insertion occurs during the natural translation process, which involves installing an amber stop codon (UAG) at the desired position and co‐expression of an orthogonal tRNA/aminoacyl‐tRNA synthetase (aaRS) pair specific for the UAA (Nödling et al., 2019). Using the photocrosslinking UAAs (Chen et al., 2017) p‐azido‐L‐phenylalanine (Azi) and p‐benzoyl‐L‐phenylalanine (Bpa), which form covalent bonds with nearby residues within a 3–4 Å range upon UV‐light exposure, we reported that the widening of non‐pore‐lining TM interfaces as well as the nexus‐TM3 interface are critical dynamics crucial for pore opening (Najjar et al., 2025). It is of note that Azi is similar to Phe, while Bpa is bulkier. Moreover, their photochemistry is distinct with Azi exhibiting less specific photochemistry and weaker stability than Bpa as described in (Chin et al., 2002; Klippenstein et al., 2014; Knowles et al., 2002; Tanaka et al., 2008).

In addition to STIM1‐induced widening of TM‐interfaces during pore opening, hydrophobic clusters further play a role in Orai1 activation. A tight hydrophobic packing along the more extracellular part of the TM1–TM2/3 interface is crucial for relaying the gating signal from the TM2/3 ring to the pore (Yamashita et al., 2017; Yeung et al., 2020). Our finding that wetting of the TM3/TM4 interface can lead to robust pore opening suggests that hydrophobicity in this region is important for maintaining the closed channel state. This is also supported by a previous study on the Orai1 and Orai3 isoforms, which showed that a reduction in hydrophobicity along TM3 leads to greater pore opening, a mechanism that is controlled in an isoform‐specific manner (Tiffner et al., 2021c). Interestingly, the hydrophobic mutation V181F in TM3 results in loss of function (LoF) (Fahrner et al., 2018; Tiffner et al., 2021b; Tiffner et al., 2021c) remaining also inactive in the presence of STIM1, while substituting V181 by the larger Tyr and Trp shows a tendency for constitutive activity (Hopl et al., 2024). Hence, the potential joint role of hydrophobicity and steric effects caused by bulky side chains at the non‐pore‐lining TM interfaces in shaping physiological Orai1 activation is still unclear.

Intrigued by this apparent interplay between sterics and (de‐)wetting in the Orai1 TM3/TM4 interface, we set out to concisely characterize the respective relevance and balance of hydrophobicity and molecular packing around V181 and oppositely located F250/F253 conducive to Orai1 activation. Upon extensive site‐directed mutagenesis, we investigate the functional effects of aromatic substitutions—alone or paired with opposing hydrophobic or aromatic mutations—as well as of local hydrophobicity in a highly conserved region in the TM3/TM4 interface. Our results show that side chain size and hydrophobicity along the TM3/TM4 interface fine‐tune the closed state. In parallel, they modulate the extent of STIM1‐induced pore opening to prevent excessive Ca^2+^ entry.

## 2 RESULTS

### 2.1 Large aromatic side chains in TM3 or TM4 lead to STIM1 independent constitutive activation

**FIGURE 1:**
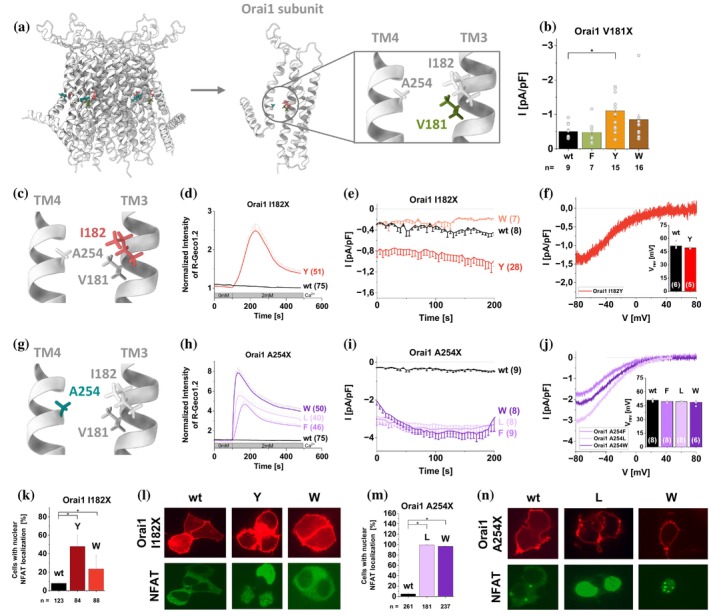
Hydrophobic or aromatic single amino acid substitutions at V181, I182, and A254 within the TM3/TM4 interface lead to gain‐of‐function. (a) Schematics showing a side view of the Orai1 channel complex and the Orai1 subunit. The inset depicts the TM3/TM4 interface surrounding V181 which is highlighted. (b) Bar graph showing the mean maximal current densities of V181F/Y/W mutations compared to Orai1 wildtype (wt) in the absence of STIM1. (c, g) Schematics of a section of the TM3/TM4 interface of an Orai1 subunit highlighting I182 (c) and A254 (g) are highlighted. (d, h) Normalized R‐GECO1.2 intensity of Orai1 mutant I182Y (d) and A254F/L/W (h) compared to Orai1 wt in the absence of STIM1. (e, i) Time courses of current densities of Orai I182Y/W (e) and Orai1 A254F/L/W (i) compared to Orai1 wt in the absence of STIM1. (f, j) Current/voltage (I/V) relationships of the Orai mutants I182Y (f) and A254F/L/W (j) in the absence of STIM1 taken at maximum current density. (k–n) NFAT translocation from the cytosol to the nucleus in YFP‐Orai1 and CFP‐NFAT co‐expressed cells in the presence of 2 mM Ca^2+^ solution. Bar charts showing the percentage of cells presenting NFAT translocation for the Orai1 mutants I182F/Y/W (k) and A254L/W (m), both compared to Orai1 wt and in the absence of STIM1. Representative images of the YFP‐Orai1 mutants I182F/Y/W (l) and A254L/W (n) and Orai1 wt are shown, together with the respective CFP‐NFAT image. Welch‐ANOVA was employed for statistical analyses of the bar graphs shown in the figure. Differences at *p* < 0.05 were considered significantly different and marked in the graph with an asterisk.

Our previous studies (Hopl et al., 2024; Najjar et al., 2025; Tiffner et al., 2021b; Tiffner et al., 2021c) have revealed that de‐wetting of the TM3/TM4 interface, particularly around V181 (Figure 1a), contributes to the maintenance of the closed state of Orai1. Unexpectedly, Orai1 V181Y showed modest yet significantly enhanced constitutive activity compared with wild type. Orai1 V181W exhibited a similar trend in the absence of STIM1. In contrast, Orai1 V181F showed no activity under the same conditions (Figure 1b) (Hopl et al., 2024). We speculated that the observed trend for constitutive activity with the larger aromatic amino acids is a size effect rather than a hydrophobicity effect, which would be in line with our findings in Najjar et al (Najjar et al., 2025). Our hypothesis together with our previous findings (Najjar et al., 2025) prompted us to investigate in more detail to what extent the side‐chain size influences the level of constitutive activity at this critical position and its surrounding. We further focused on two other positions (I182, A254) in the immediate vicinity of V181 (Figure 1a) and found that they also play an important role in maintaining the closed Orai1 state. Replacement of I182 (Figure 1c) with the larger aromatic tyrosine (I182Y) leads to a significantly higher intracellular Ca^2+^ concentration in Ca^2+^ imaging studies (Figure 1d) and constitutive activity in electrophysiological experiments (Figure 1e) compared to Orai1 I182W and Orai1 wt in the absence of STIM1. Similar to Orai1 V181K and other V181‐GoF‐mutants (Hopl et al., 2024; Tiffner et al., 2021b), Orai1 I182Y shows an inward rectifying current/voltage (I/V) relationship with a reversal potential (V_rev_) in the range of +50 mV (Figure 1f), similar to typical CRAC channel currents (Hopl et al., 2024; Peinelt et al., 2006; Prakriya et al., 2006). Similarly, A254 in TM4 (Figure 1g), located directly opposite V181 and I182, showed enhanced intracellular Ca^2+^ concentrations and constitutive currents in the absence of STIM1 when substituted by amino acids with larger side chain size (A254L/F/W) (Figure 1h,i). Again, these constitutive mutants displayed a V_rev_ of about +50 mV (Figure 1j) resembling Orai1 wt currents (Feske et al., 2006; Vig et al., 2006b) activated by STIM1. Overall, the size effect seems to dominate the hydrophobic effect previously described (Hopl et al., 2024; Tiffner et al., 2021c) to maintain the closed state.

In the presence of STIM1, Orai1 I182Y and Orai1 A254L tended to yield current levels slightly lower compared to Orai1 wild‐type, while Orai1 A254F/W showed enhanced activation, although none of the effects reached statistical significance (Figure S1A,B). These variations may reflect a more complex involvement of steric and hydrophobicity along the TM3/TM4 interface.

To assess the impact of these mutants on downstream signaling, we analyzed the translocation of NFAT (Nuclear Factor for the Activation of T‐cells). NFAT is dephosphorylated by calcineurin in a process that depends on the intracellular Ca^2+^ concentration, leading to its translocation from the cytosol to the nucleus (Li et al., 2007; McCaffrey et al., 1993). To monitor this process, we co‐expressed fluorescently labeled NFAT and investigated its translocation to the nucleus using confocal fluorescence microscopy. In accord with the constitutive activity of the Orai1 I182Y and Orai1 A254L/W mutants, we found significantly increased NFAT translocation, whereas drastically reduced NFAT translocation was observed in the presence of Orai1 wt alone or Orai1 I182W (Figure 1k–n).

In conclusion, substitutions by aromatic amino acids in the TM3‐TM4 interface around V181 can lead to constitutively active Orai1 channels which can induce downstream signaling as shown via NFAT translocation. We propose that the size rather than the hydrophobic character of the aromatic amino acids at the tested positions is the dominant determinant mediating pore opening, even though hydrophobicity has previously been implicated in stabilizing the inactive, closed state (Hopl et al., 2024; Tiffner et al., 2021c).

### 2.2 Double substitutions combining different hydrophobic substitutions in TM3 and TM4 can elevate constitutive activity

**FIGURE 2:**
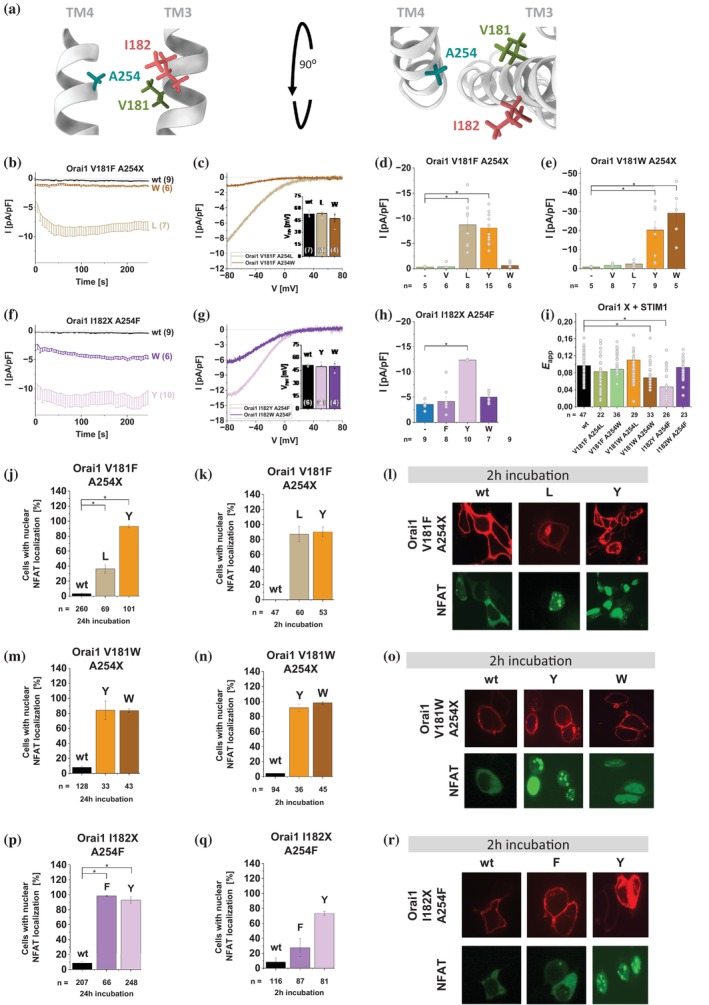
Double mutants combining different hydrophobic amino acid substitutions in TM3 and TM4 can elevate constitutive activity. (a) Schematics showing a side and a top view of a section of the TM3/TM4 interface surrounding V181 of an Orai1 subunit. The observed positions V181, I182, and A254 are highlighted. (b, f) Time courses of current densities of the mutants V181F A254W/L (b) and I182Y/W A254F (f) compared to Orai1 wt in absence of STIM1. (c, g) I/V relationship of current densities of Orai1 mutants V181F A254W/L (c) and I182Y/W A254F (g) in absence of STIM1. (d, e) Bar graphs showing maximum current densities of Orai1 mutants V181F A254V/L/Y/W (d), V181W A254V/L/Y/W (e), and I182F/Y/W A254F (h) with increasing side chain size (with V < L < Y < W and F < Y < W) compared to V181F, V181W, and A254F, respectively, in absence of STIM1. (i) Bar graphs of maximal *E*_app_ from time course FRET experiments at *t* = 480 s of STIM1‐CFP and Orai1‐YFP mutants V181F A254L/W, V181W A254L/W and Orai1 I182Y/W A254F compared to Orai1 wt; store depletion was induced by 1 μM thapsigargin (TG). (j, m, p) Bar graphs showing the percentage of cells with nuclear NFAT translocation for the Orai1 mutants V181F A254V/L/Y (j), V181W A254V/Y/W (m), and I182F/Y A254F (p) compared to Orai1 wt in the absence of STIM after 24 h incubation with 2 mM Ca^2+^. (k, n, q) Bar graphs showing the percentage of cells with nuclear NFAT translocation for the Orai1 mutants V181F A254L/Y (k), V181W A254V/W/Y (n), and I182F/Y/W A254F (q) compared to Orai1 wt in the absence of STIM after 2 h incubation with 2 mM Ca2+. (l, o, r) Representative images of the YFP‐Orai1 mutants V181F A254L/Y (l), V181W A254V/Y/W (o), and I182F/Y/W A254F (r) and Orai1 wt are shown, together with the respective CFP‐NFAT image. Welch‐ANOVA was employed for statistical analyses of the bar graphs shown in the figure. Differences at *p* < 0.05 were considered significantly different and marked in the graph with an asterisk.

While the substitution of single amino acids by larger aromatic side chains in the plane of V181 on both sides of the TM3/TM4 interface can lead to weak constitutive activity, an additional substitution by a hydrophobic or aromatic amino acid at an opposite position (Figure 2a) further enhanced the GoF activity in several cases, independent of STIM1 (Figures 2b–h and S1C–I). Among the combinations tested, Orai1 V181F with A254L or A254Y produced a significantly increased current density both without and with STIM1, even before store‐depletion unlike the inactive Orai1 V181F (Figures 2b–d and Figure S1C). Other combinations of Orai1 V181F with A254V/W and F253Y/W did not induce constitutive activation (Figures 2d and S1C,D). Regarding the weakly active Orai1 V181W, we showed that a combination with A254Y/W (Figures 2e and S1E) or F253W (Figure S1F) resulted in significantly enhanced currents without STIM1 co‐expression or before store‐depletion in the presence of STIM1. In contrast, Orai1 V181W combined with A254V/L and F253Y did not significantly enhance current densities compared to Orai1 V181W (Figures 2e and S1E,F). Additionally, the constitutive activity of Orai1 A254F was significantly enhanced in combination with I182Y (Figures 2f–h and S1G) or V181W (Figure S1H), but not I182F/W (Figures 2f–h and S1G). Despite our findings that some combinations of hydrophobic and/or aromatic side chains lead to constitutive activity, we were unable to establish a clear correlation between the volume of the side chains and the current density values. This is presumably due to amino acids adopting different orientations in the channel complex. Despite the changes in activity levels, V_rev_ of the constitutively active double mutants was in the range of +40 and +50 mV, comparable to V_rev_ of Orai1 wt induced by STIM1, as exemplified by Orai1 V181F A254L/W or Orai1 I182W/Y A254F (Figure 2c,g).

In the presence of STIM1, most double mutants (Orai1 V181F A254L, Orai1 V181W A254Y/W, Orai1 V181W F253W, Orai1 I182Y A254F, Orai1 V181W A254F) that are already highly constitutively active without STIM1 (~10–20 pA/pF) did not show a significant increase in current density after store depletion, while double mutants with smaller constitutive currents (Orai1 V181F A254W, Orai1 I182W A254F) exhibited a significant increase in current density in the presence of STIM1 (Figure S1C–K). Interestingly, some double mutants (e.g., Orai1 V181F A254V, Orai1 V181W A254V/L, Orai1 V181F F253Y/W, Orai1 V181W F253Y, Orai1 I182F A254F) showed little or no increase in current density in the presence of STIM1 (Figure S1C–G).

Since several highly constitutively active double mutants do not show further enhancement in current upon store‐depletion in the presence of STIM1 (Figure S1C–K), we examined a selection of double mutants for STIM1 binding. Orai1 V181F A254L/W‐YFP mutants showed a slight, non‐significant reduction in Förster resonance energy transfer (FRET) with CFP‐STIM1 (Figure 2i). In contrast, Orai1 V181W A254L‐YFP exhibited slightly enhanced FRET with CFP‐STIM1, while Orai1 V181W A254W‐YFP displayed significantly reduced FRET with CFP‐STIM1. Further FRET experiments revealed a comparable FRET of STIM1‐CFP with Orai1 I182W A254F‐YFP like with Orai1 wt‐YFP, while FRET with Orai1 I182Y A254F‐YFP was drastically reduced (Figure 2i).

We next assessed downstream signaling by examining NFAT nuclear translocation in cells overexpressing our constitutively active, electrophysiologically tested double mutants combining aromatic with hydrophobic or aromatic residues. NFAT nuclear accumulation largely mirrored the electrophysiological results (Figure 2j–r). Specifically, in accord with the constitutive activity of Orai1 V181F A254L/Y, Orai1 V181W A254Y/W and Orai1 I182F/Y A254F, we also observed enhanced NFAT translocation for cells overexpressing the respective mutants (Figure 2j,m,p). Since NFAT levels after 24 h incubation in 2 mM Ca^2+^ solution (Figure 2j,m,p) did not fully correlate with the recorded current densities (Figure 2d,e,h) potentially due to side effects of drastically enhanced Ca^2+^ levels, we examined NFAT translocation after a reduced incubation time (2 h). As expected, the extent of NFAT translocation after 2 h (Figure 2k,n,q) showed a better correlation with the constitutive currents of the V181 mutant combinations (V181F A254L/Y; V181W A254Y/W, Orai1 I182F/Y A254F; Figure 2j–r). Orai1 I182F A254F, which showed weak constitutive activation in electrophysiology (Figure 2h), also showed NFAT translocation more comparable to cells with Orai1 wt (Figure 2q). To confirm the specificity of NFAT translocation to Orai1 wt/mutant function, we applied the Orai1 specific blocker CM‐4620 (Stauderman, 2018; Waldron et al., 2019; Whitten, 2011) on cells containing the constitutively active mutants Orai1 I182F/Y A254F and the NFAT reporter. Both mutants showed significantly reduced NFAT translocation compared to the absence of the CM‐4620 (Figure S1L), indicating clear dependence of NFAT translocation on Orai1 activity.

Overall, several double substitutions combining aromatic and/or hydrophobic mutations in the plane of V181 of the TM3/TM4 interface led to robust constitutive activation that was not further enhanced by STIM1. Aromatic/hydrophobic double substitutions leading to high constitutive activity may impact STIM1‐coupling in some cases, which could explain the lack of further increase in store‐operated current levels. Constitutive activity correlates with the extent of NFAT translocation into the nucleus.

### 2.3 Orai1 V181F is retained in the closed state due to an inhibitory role of the V181F‐F250 contact

**FIGURE 3:**
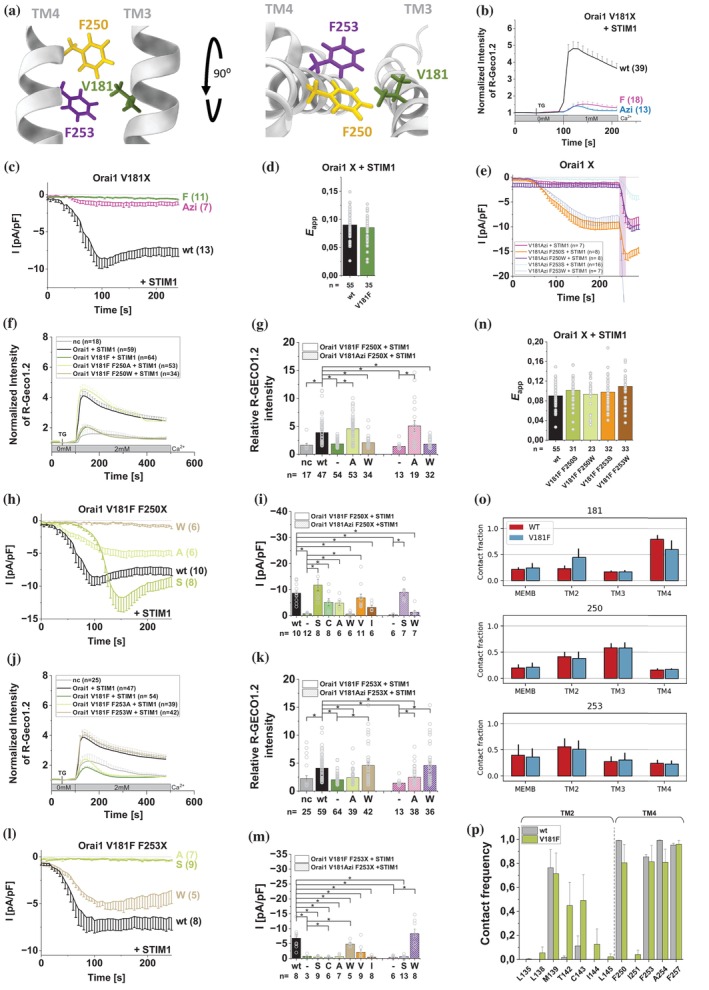
Phe at V181 in TM3/TM4 interface triggers loss‐of‐function due to an inhibitor interplay with F250. (a) Schematics of the section of the Orai1 TM3/TM4 interface, surrounding V181. Relevant positions V181, F250, and F253 are highlighted. (b) Relative R‐GECO1.2 intensities of V181Azi/F in the presence of STIM1, compared to Orai1 wt. (c) Time course of the current densities of V181Azi/F in presence of STIM1, compared to Orai1 wt. (d) Bar diagram of maximal *E*_app_ from FRET experiments at *t* = 480 s of STIM1‐CFP and Orai1‐YFP mutant V181F compared to Orai1 wt; store depletion was induced by 1 μM thapsigargin (TG). (e) Time courses of current densities of Orai1 V181Azi F250S/W and Orai1 V181Azi F253S/W compared to Orai1 V181Azi in the presence of STIM1 respectively. The purple bar represents the illumination with UV‐light (365 nm) for 15 s. (f, j) Relative R‐GECO1.2 intensities of Orai1 mutants V181F F250A/W (f) and V181F F253A/W (j) mutants compared to V181F, Orai1 wt and untransfected cells (negative control; nc) in the presence of STIM1. (g, k) Bar graph showing maximum relative R‐GECO1.2 intensities of Orai1 mutants V181Azi/F F250A/W (g) and V181Azi/F F253A/W (j) compared to V181Azi/F respectively, Orai1 wt, and nc, in the presence of STIM1. (h, l) Time courses of current densities of Orai1 mutants V181F F250A/S/W (h) and V181F F253A/S/W (l) in presence of STIM1 compared to Orai1 wt. (i, m) Bar graphs showing maximum current densities of Orai1 V181F F250S/C/A/W/V/I and Orai1 V181Azi F250S/W (i) and Orai1 V181F F253S/C/A/W/V/I and Orai1 V181Azi F253S/W (m) in the presence of STIM1 compared to Orai1 V181F/ V181Azi respectively and to Orai1 wt. (n) Bar graphs of maximal *E*_app_ of FRET experiments at *t* = 480 s between STIM1‐CFP and Orai1‐YFP mutants V181F F250S/W and V181F F253S/W compared to V181F and Orai1 wt. (o) Inter‐domain contacts in the WT and V181F mutant for residues V/F181, F250, and F253. Contact fractions denote the proportion of all contacts formed by a residue that involve a given transmembrane domain or the membrane. (p) Contact frequencies between residue V/F181 and its partners in TM2 and TM4, averaged over all subunits. Contact frequency reflects how often a contact was detected across the simulation. Welch‐ANOVA was employed for statistical analyses of the bar graphs shown in the figure. Differences at *p* < 0.05 were considered significantly different and marked in the graph with an asterisk.

The single substitution V181F alone or combined with mutations at opposite positions (Orai1 V181F A254V, Orai1 V181F F253Y/W) led to almost or completely inactive Orai1 variants in the absence as well as in the presence of STIM1 (Figures 1b and S1C,D). To investigate the reasons for the inactivity of Orai1 V181F, we conducted further studies with an analogous mutant in which the photocrosslinking UAA 4‐azido‐L‐phenylalanine (Azi) was introduced at position V181 (Figure 3a–c). Azi possesses similar side chain properties to Phe and Tyr (Coin et al., 2011; Kurttila et al., 2021; Lang & Chin, 2014; Lim et al., 2015), but exhibits enhanced polarity due to the azide group with a dipole moment of ~2 Debye (Bergmann & Schütz, 1931; Smith et al., 2020; Wolfshorndl et al., 2012) (see Table S1). As with the Orai1 V181F mutant, Orai1 V181Azi remains almost inactive regardless of the presence of STIM1 (Figure 3b,c). Thus, the increased polarity of Azi compared to Phe is not sufficient to restore store‐operated activation. STIM1 coupling is retained for both Orai1 V181F (Figure 3d) and Orai1 V181Azi as revealed by FRET value comparable to Orai1 wt upon store‐depletion (Najjar et al., 2025). Upon additional UV‐light exposure, Orai1 V181Azi bound to STIM1 is activated (Figure 3e), indicating that the signal propagation from TM3 to the pore remains essentially intact. The photochemical reaction of Azi involves the photocleavage of molecular nitrogen, which typically persists for a few nanoseconds (Leyva et al., 2022). Our density functional theory (DFT) calculations reveal a dipole moment of 4.42 of the excited singlet state (Table S2) indicating reduced hydrophobicity compared to the Azi's non‐excited state. In the absence of a suitable reactive site nearby, it converts into a less reactive but stable ketenimine, while otherwise it forms a covalent bond with a nearby partner (C‐H, O‐H, N‐H) (Budyka, 2007; Gritsan & Platz, 2009; Leyva et al., 2022). These chemical reactions occur within the ns range (Aydin & Coin, 2023; Leyva et al., 2022). Given that UV‐induced functional changes (10–15 s) occur on a timescale approximately 10‐fold longer than the lifetime of excited Azi (ns) and in view of the robustness, high reproducibility and specificity of UV effects, we attribute the UV‐induced functional effects to structural changes upon photocrosslink formation with a nearby residue rather than local changes in hydrophobicity (Najjar et al., 2025). In contrast to Azi at V181, which does not allow store‐dependent activation, we recently reported that the bulkier photocrosslinking UAA Bpa at position V181 (Orai1 V181Bpa) allowed normal store‐operated activation by STIM1. UV light exposure further enhanced Orai1 V181Bpa currents (Najjar et al., 2025).

A detailed examination of the Orai1 homology model based on the crystal structure of the closed state of dOrai (PDB: 4HKR) revealed that residues F250 and F253, located at opposing positions in TM4, should be in range to directly interact with V181F/Azi (Figure 3a). We conjectured that the removal of the bulky aromatic side chains at position 250 or 253 could restore STIM1‐mediated activation. We thus examined the effects of V181F in combination with amino acid substitutions with different properties at F250 or F253, respectively.

Initially, we started investigating the impact of different substitutions of F250 in Orai1 V181F using Ca^2+^‐imaging. These experiments showed that STIM1 and Orai1 V181F F250W co‐expressing cells exhibited comparably low store‐operated Ca^2+^ level enhancements similar to cells containing Orai1 V181F or mock transfected cells. In contrast, Orai1 V181F F250A in the presence of STIM1 led to Ca^2+^ levels comparable to Orai1 wt (Figure 3f,g). In electrophysiological experiments, we compared the effects of different F250X substitutions (X = S/C/A/W/V/I) inserted in Orai1 V181F. While F250S/C/A/V restored STIM1‐mediated current levels of Orai1 V181F to values comparable to or even higher than those of STIM1‐mediated Orai1 wt currents, Orai1 V181F F250I showed lower and Orai1 V181F F250W no current activation (Figure 3h,i). In agreement with these findings, STIM1‐mediated activation of the UAA‐containing mutant Orai1 V181Azi F250S/A was again able to overcome the inhibitory effect of V181Azi, while Orai1 V181Azi F250W, similar to Orai1 V181Azi, showed hardly any store‐operated activaiton (Figure 3g,i).

Replacing F253 in Orai1 V181F with either large hydrophobic or small, less hydrophobic amino acids appeared to have the opposite effects to those of F250 mutants. Ca^2+^ imaging studies revealed that the insertion of a Trp (V181F/Azi F253W) was able to overcome the inhibition of V181F/Azi and led to STIM1‐mediated Ca^2+^‐levels comparable to wt (Figure 3j,k). The smaller less hydrophobic Ala at position 253 in Orai1 V181F was unable to increase intracellular Ca^2+^‐levels beyond those of Orai1 V181F in the presence of STIM1 (Figure 3j,k). Electrophysiological experiments with different substitutions at F253 in combination with V181F (V181F F253S/C/A/W/V/I) showed that only F253W and F253V (V181F F253W/V) were able to increase STIM1‐mediated current densities compared to Orai1 V181F (Figure 3l,m). Similarly, the analog mutant Orai1 V181Azi F253W showed store‐operated activation, while Orai1 V181Azi F253S/A remained inactive in the presence of STIM1 (Figure 3k,m).

We further examined a selection of UAA‐containing double mutants (Orai1 V181Azi F250S/W, Orai1 V181Azi F253S/W) for their response to UV exposure upon maximal STIM1‐mediated activation (Figure 3e). The STIM1‐activatable mutants Orai1 V181Azi F250S and Orai1 V181A F253W showed additional current enhancements reaching higher levels upon UV exposure than STIM1‐activated Orai1 V181Azi. Notably, the inactive Orai1 V181Azi F250W and Orai1 V181Azi F253S also exhibited UV‐induced current enhancements in the presence of STIM1 (Figure 3e). Hence, UV‐induced activation despite LoF indicates that signal propagation to the pore and thus, typical CRAC‐channel activation can be recovered in these mutants.

To ensure that the alterations in Ca^2+^‐levels or current density were not influenced by a change in binding behavior between STIM1 and the respective Orai1 mutants, we performed FRET microscopy experiments. FRET value obtained upon store‐depletion induced by TG (1 μM) confirmed that neither V181F nor combinations of V181F with F250S/W or F253S/W changed the binding efficiency compared to Orai1 wt (Figure 3n).

To improve our understanding of the functional effects of the F substitutions we performed all‐atom MD simulations of Orai1 and Orai1 V181F to assess the contacts of positions V181/F181, F250, and F253 with TM2, TM3, and TM4 as well as their interactions with the PM. In Orai1 we found that V181 mostly forms contacts with residues in TM4 (79%), while only 23% of contacts involve residues in TM2. More than 50% of the contacts formed by F250 are with residues in TM3, and 41% are with residues in TM2. In contrast, F253 forms more than 50% of its contacts with TM2 and only 27% of its contacts with TM3. Additionally, F253 has a larger fraction of its contacts with the membrane than F250, indicating different preferred interaction partners of the two residues. Overall, F250 favors interactions with TM3, whereas F253 favors TM2. Moreover, we compared contact probabilities for Orai1 wt and Orai1 V181F. Compared to V181 in Orai1 wt, a redistribution of contacts can be observed for F181, with 44% of its contacts involving TM2 and 60% involving TM4 (Figure 3o,p). The larger standard deviation of contact fractions in Orai1 V181F compared to Orai1 wt indicates that F181 experiences a more dynamic local environment. F181 showed a slight shift in contact preferences compared to V181, whereas F250 and F253 were unchanged in the mutants relative to wt; this shift is also reflected in our analysis of contact residues of V181 and F181, respectively. V181 finds most contact partners in TM4 and only M139 as a dominant contact partner in TM2. In contrast, F181's contacts shift toward TM2, specifically the TM2 of the next subunit (TM2′), favoring T142 and C143, and contacts with F250 and A254 in TM4 are weaker (Figure 3o). We therefore propose that the TM3–TM4 contact formed by F181–F250 plays a predominant role in establishing inhibitory interactions, while the impact of F253 in maintaining the inactive state is less profound. The recovery of Orai1 V181Azi F253W function suggests that steric effects at position 253 can modulate channel activity. Moreover, the enhanced contacts formed by V181F with TM2 may help to stabilize the channel in the closed state (Figure 3o,p).

Our functional results suggest that LoF of Orai1 V181F results from an inhibitory interplay of F181 and F250, which is supported by our MD simulations showing higher contact fractions with TM3 compared to TM2 for F250. Moreover, the MD simulations indicate an enhanced interaction of V181F with TM2, which may have an additional inhibitory impact.

### 2.4 Hydrophobic core along the TM3/TM4 interface maintains the closed state

**FIGURE 4:**
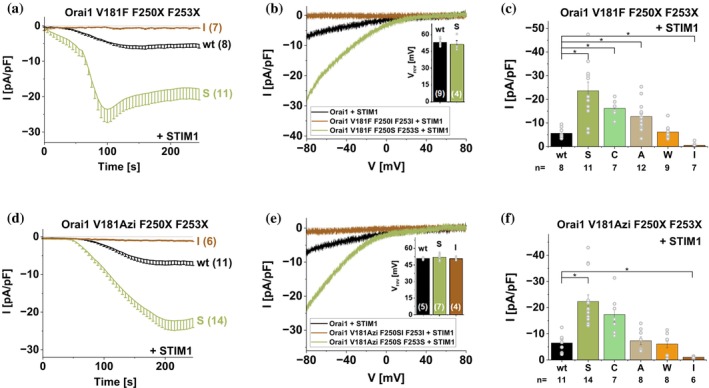
Hydrophobic core around V181F maintains closed state of Orai1. (a, d) Time course of current densities of Orai1 mutants V181F F250S/I F253S/I (a) and V181Azi F250S/I F253S/I (d) in the presence of STIM1 compared to Orai1 wt. (b, e) I/V relationships of V181F F250S/I F253S/I (b) and V181Azi F250S/I F253S/I (e) in the presence of STIM1 taken at maximum current levels. (c, f) Bar graph of the mean maximum current densities of Orai1 mutants V181F F250S/C/A/W/I F253S/C/A/W/I (c) and V181Azi F250S/C/A/W/I F253S/C/A/W/I (f) with increasing side chain hydrophobicity (S < C < A < W < I) in the presence of STIM1 compared to Orai1 wt. Welch‐ANOVA was employed for statistical analyses of the bar graphs shown in the figure. Differences at *p* < 0.05 were considered significantly different and marked in the graph with an asterisk.

We further investigated the effects of double Phe substitutions in TM4 around V181F/Azi at the TM3/TM4 interface. Substitutions at F250 and F253 ranged from hydrophilic to hydrophobic ones, including Ser, Cys, Ala, Trp and Ile (V181F/Azi F250S/C/A/W/I F253S/C/A/W/I) according to Eisenberg et al (Eisenberg et al., 1984). We chose the Eisenberg hydrophobicity scale due to its ability to evaluate hydrophobicity and amphipathic properties, which are critical determinants for protein stability and membrane integration (see further details in Table S3) (Eisenberg et al., 1984). Electrophysiological experiments on Orai1 V181F/Azi F250S/C F253S/C did not only show that the inhibitory effect of V181F was overcome, but also that the current density even increased significantly, by a factor 3–4, compared to those of Orai1 wt (Figures 4a–f and S2A,B). The replacement of both by Ala or Trp (V181F/Azi F250A/W F253A/W) still overcame the inhibitory effect of V181F and led to slightly higher or comparable current densities to those of Orai1 wt, respectively (Figure 4a–f). However, replacing F250 and F253 by Ile (V181F/Azi F250I F253I) completely inhibited STIM1‐induced activation of the Orai1 mutant (Figure 4a–f and S2A). Furthermore, V_rev_ for these triple mutants was between +40 and +50 mV, as exemplarily shown for Orai1 V181F F250S/I F253S/I and Orai1 V181Azi F250S/I F253S/I. This indicates that the selectivity of the mutant channels was not altered (Figure 4b,e).

Correlating hydrophobicity in Orai1 triple mutants at positions 250 and 253 with STIM1‐mediated current densities (Orai1 V181F/Azi F250X F253X; X = S, C, A, W, I) revealed a near‐perfect Pearson correlation using the Eisenberg scale (V181F: 0.99; V181Azi: 0.96; see for further detail in Table S4) (Eisenberg et al., 1984; Koehler et al., 2009; Kyte & Doolittle, 1982; Roseman, 1988). In contrast, van der Waals volume (V_vdW_) and side‐chain size exhibit weaker correlations with current densities of Orai1 V181F/Azi triple mutants (Tables S4, S5).

To further corroborate that the double substitution F250S F253S in Orai1 V181F/Azi is required to strongly enhance current densities, we tested the effect of F250S F253W and F250W F253S in V181Azi. Both exhibited store‐operated current activation; however, to levels lower than that of Orai1 V181Azi F250S F253S, highlighting the critical role of hydrophobicity in regulating the activation levels (Figure S2B).

Beyond Azi, we evaluated the impact of Bpa: a more voluminous, hydrophobic photocrosslinking UAA. Orai1 V181Bpa combined with analog substitutions of F250 and F253, as reported above, enables store‐operated or even constitutive activity (constitutive in particular for V181Bpa F250X F253X with X = S, A, C, L, Y; Figure S2E,F). However, the correlation of current densities with any of the hydrophobicity scales was low compared to the V181F/Azi triple mutants (SI). Among the triple mutants, only Orai1 V181Bpa F250Y/W F253Y/W showed higher STIM1‐mediated currents compared to V181Bpa alone, suggesting an effect of the side chain size on the current density (Figure S2E,F). Indeed, correlation analysis of only hydrophobic residues starting from Leu with store‐operated current densities revealed a near‐perfect Pearson correlation coefficient, highlighting a dominant role of side chain size over hydrophobicity under these conditions (Table S5).

Overall, a gradual increase in the hydrophobicity of the substitutions for F250 and F253 (S < C < A < W < I; Kyte & Doolittle, 1982) within V181F/Azi led to a stepwise decrease in the resulting STIM1‐induced current density (Figure 4c,f). Ser‐substitution in Orai1 V181F even allowed to exceed STIM1‐induced current densities of Orai1 wt. The success of the Eisenberg scale which models solvation effects of simple peptide systems and globular proteins (Eisenberg et al., 1984) may also be related to the change of the chemical environment of V181 during activation of Orai1 (see Discussion section). Furthermore, drastically enhanced side chain size, as exemplified with Bpa mutants, also allows higher STIM1‐mediated current activation than under wt conditions.

The dominant effect of bulky side chains over hydrophobicity on current levels is particularly pronounced for UV‐induced currents. Most Azi‐containing mutants, regardless of their activity in the presence of STIM1, exhibited increased current density after UV irradiation, indicating that channel function in response to this distinct stimulus remained intact (Figure S2A–D). While no correlation of hydrophobicity with UV‐induced current size was detected, it is clearly visible that hydrophobic amino acid substitutions with increasing side chain size starting from Leu correlate with an increase in UV‐induced current densities, as confirmed by a near‐perfect Pearson correlation coefficient for these conditions (Tables S4, S5). Moreover, STIM1‐activated Orai1 V181Azi F250W/S F253S/W showed drastically enhanced UV‐induced current densities, further supporting the additional impact of amino acid side chain size along the TM3/TM4 interface on current activation. Under conditions using Bpa instead of Azi, UV light did not further strongly enhance STIM1‐mediated currents (Figure S2E,F). Correlation analysis revealed drastically reduced Pearson correlation coefficients for hydrophobicity with current densities of Azi‐ and Bpa‐mutants using Eisenberg scale (SI). In contrast, a stronger correlation of V_VdW_ volume with current densities of the UAA mutants was detected. Most prominently, a near‐perfect correlation was detected for V_VdW_ of V/L/Y/F/W substitutions with currents of Bpa‐triple mutants before and after UV exposure (Figure S2F [gray shaded area; Table S5]).

Notably, Orai1 V181Azi F250C/S F253C/S showed weak or no UV‐induced activation, despite significantly enhanced STIM1‐mediated currents amounting above wt levels (Figure S2A–D). These strongly enhanced currents might be assigned to STIM1‐induced conformational arrangements impairing further UV‐induced functional changes in accord with our previous findings in Najjar et al (Najjar et al., 2025).

In summary, we found a gradual decrease in current density with increasing hydrophobicity in the area surrounding V181, marking hydrophobicity as main contributing factor for the maintenance of the closed state and fine‐tuning of STIM1 induced maximal activity of Orai1. As soon as the interface widens more drastically by the integration of bulky amino acids (e.g., Bpa) in this area, current activation was correlated with side chain size, in accord with our previous findings (Najjar et al., 2025).

### 2.5 Decreased hydrophobicity in the TM3/TM4 interface in the plane of V181 leads to STIM1 independent Orai1 activation

**FIGURE 5:**
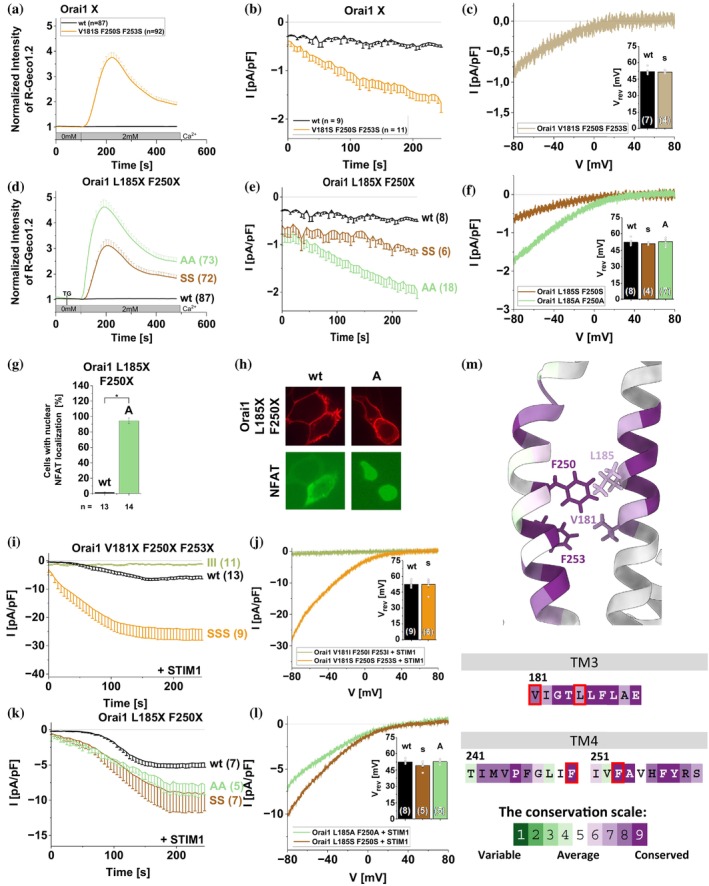
Decrease of hydrophobicity in the plane of V181 in the TM3/TM4 interface leads to constitutive Orai1 activity. (a, d) Relative R‐GECO1.2 intensities of Orai1 mutants V181S F250S F253S (a) and L185A/S F250A/S (d) compared to Orai1 wt, in the absence of STIM1. (b, e) Time courses of current densities of Orai1 mutants V181S F250S F253S (b), and L185A/S F250A/S (e) compared to Orai1 wt in the absence of STIM1. (c, f) I/V relationships of current densities of the Orai1 mutants V181S F250S F253S (c) and L185A/S F250A/S (f) in the absence of STIM1. (g) Percentage of cells with nuclear NFAT translocation for Orai1 mutants L185A/S F250A/S in the absence of STIM1. (h) Representative images of cells expressing the Orai1 mutants L185A/S F250A/S and Orai1 wt labeled with YFP and the corresponding images of CFP‐NFAT. (i, k) Time courses of current densities of Orai1 mutants V181S/I F250S/I F253S/I (i) and L185A/S F250A/S (k) in the presence of STIM1 compared to Orai1 wt. (j, l) I/V relationships of current densities of the Orai1 mutants V181I/S F250I/S F253I/S (j) and L185A/S F250A/S (l) in the presence of STIM1. (m) Conservation analysis of Orai1 using ConSurf‐DB (a database of precalculated ConSurf evolutionary conservation profiles for proteins of known structure). Amino acid sequence including the relevant positions V181, L185, F250, F253, and F257 (highlighted in red), color‐coded from “Variable” (dark green) to “Conserved” (dark purple). Positions are highlighted in the corresponding color in the schematic above. Welch‐ANOVA was employed for statistical analyses of the bar graphs shown in the figure. Differences at *p* < 0.05 were considered significantly different and marked in the graph with an asterisk.

As Phe residues in TM4 appear to govern the degree of STIM1‐dependent Orai1 pore opening, we further investigated their influence together with hydrophobic counterparts in TM3 in the absence of the V181F background. Specifically, in addition to F250 and F253, we replaced V181 with Ser to further lower hydrophobicity at the TM3/TM4 interface (V181S F250S F253S). This triple mutant showed weak constitutive activity in Ca^2+^ imaging and electrophysiological recordings (Figure 5a,b). In addition to V181, we also analyzed another hydrophobic contact, L185‐F250, which is one helical turn upstream of V181 in TM3. The reduction of hydrophobicity between L185 and F250 by exchange with Ser (L185S F250S) or Ala (L185A F250A) also resulted in STIM1‐independent weak constitutive activity in both cases (Figure 5d,e). All three mutants exhibited a strongly inward rectifying I/V relationship with a V_rev_ between +40 and +50 mV, thus matching the biophysical characteristics of STIM1 activated Orai1 wt (Figure 5c,f). Analogously, in STIM1/STIM2 knock‐out HEK293 cells we detected constitutive activity of Orai1 V181S F250S F253S and Orai1 L185S/A F250S/A with an inward rectifying I/V relationship and a V_rev_ around +50 mV (Figure S3A,B).

We further tested whether the downstream signaling of NFAT translocation reflects the constitutive activity of L185A F250A. Indeed, the magnitude of the current levels of constitutively active mutants was reflected by the extent of NFAT translocation into the nucleus. The constitutively active Orai1 L185A F250A mutant also showed high translocation rates of NFAT (Figure 5g,h).

Next, we examined Orai1 V181S F250S F253S and Orai1 L185A/S F250A/S in the presence of STIM1. The STIM1‐mediated current levels of the triple mutant exceeded those of Orai1 wt. Vice versa, an increased hydrophobicity along the interface by replacing V181, F250, and F253 with Ile (V181I F250I F253I) completely abolished currents compared to wt (Figure 5i). Despite the greatly increased current densities of Orai1 V181S F250S F253S mediated by STIM1, fast Ca^2+^ dependent inactivation (FCDI) remained unaltered compared to Orai1 wt (Figure S3C). The constitutively active mutants Orai1 L185A F250A and Orai1 L185S F250S showed slightly, but not significantly, enhanced currents compared to Orai1 wt when co‐expressed with STIM1. This is likely due to the retained presence of other hydrophobic amino acids close to the cytosol (e.g., V181, F253) (Figure 5k). In accord with the effects the mutants showed in the absence of STIM1, in the presence of STIM1 all three exhibited strongly inward rectifying I/V relationships with a V_rev_ between +40 and +50 mV, unchanged from STIM1‐mediated currents of Orai1 wt (Figure 5j,l).

We showed above, that hydrophobic residues near V181 help maintain the closed state of the channel and prevent overactivation by STIM1, as shown by the Orai1 V181S F250S F253S mutant in the presence of STIM1. Motivated by this, we used ConSurf‐DB (Ben Chorin et al., 2020) to assess residue conservation in the TM3/TM4 interface (aa 181–190, aa 241–260). Consurf‐DB provides pre‐calculated evolutionary conservation profiles for proteins of known structure in the Protein Data Bank (PDB). The conservation analysis showed the highest conservation scores for F250, F253, and F257, the second highest score for V181, and the third highest score for L185 (Figure 5m). These findings indicate great functional relevance of these residues at these positions throughout the course of evolution.

Overall, the reduction of hydrophobicity along the TM3/TM4 interface resulted in weak constitutive activity, which is greatly increased by STIM1 and exceeds the Orai1 wt levels. This underlines the crucial role of hydrophobic residues not only in maintaining the Orai1 closed state but also fine‐tuning the extent of Ca^2+^ permeation.

## 3 DISCUSSION

Our site‐directed mutagenesis analysis is focused on V181 and its environment and highlights the critical role in balancing physiological Orai1 function based on steric restraints and the hydrophobic effect along the TM3/TM4 interface. Orai1 pore opening can be achieved by increased side chain volume around V181, a mechanism likely induced by STIM1‐binding conducive to TM3/TM4 interface dilation dovetailing with our previous findings (Hopl et al., 2024; Najjar et al., 2025). In addition to this conformational rearrangement mediating Orai1 activation, the extent of Ca^2+^ influx is fine‐tuned by the hydrophobic effect along the peripheral TM3/4 domain interface. Overall, our data indicate that amino acid properties, most importantly side chain size and hydrophobicity, are precisely optimized by nature in a way that the Orai1 channel can be reliably and sufficiently activated by STIM1 while simultaneously preventing excessive activation and intracellular Ca^2+^ overload.

In contrast to the previously reported role of hydrophobicity in maintaining the closed state of Orai1 (Hopl et al., 2024; Tiffner et al., 2021c), we show that aromatic side chains at positions V181, I182, and A254 can induce weak constitutive activity, which is further enhanced by several double substitutions on the opposing helix of the TM3/TM4 interface. This behavior probably stems from the size of the amino acid having a dominant effect over hydrophobicity. This assumption aligns with our previous report (Najjar et al., 2025), where UV‐mediated activation of Orai1 mutants containing photocrosslinking UAAs at specific positions in the TM3/TM4 interface correlated with increased side chain size. Similarly, molecular dynamic (MD) simulations showed that restraining TM3 and TM4 at certain widened distances promotes pore hydration and dilation (Najjar et al., 2025). UV‐light exposure to certain Orai1‐mutants containing the UAA along TM3 (e.g., V181) showed reduced activation by STIM1 compared to when STIM1‐activation occurred before UV light irradiation (Najjar et al., 2025). This supports the notion that STIM1 coupling triggers a widening of the TM3/TM4 interface. Thus, aromatic substitutions likely induce constitutive pore opening by increasing the separation between TM3 and TM4, a structural rearrangement likely mimicked by STIM1 coupling during Orai1 wt activation (Najjar et al., 2025). In line with these findings, charged side chains at V181 or A254 induced robust constitutive activity. On the one hand, this is possible due to enhanced hydration along the TM3/TM4 interface. On the other hand, this leads to hydration‐induced swelling of the channel complex subsequently triggering Orai1 pore opening (Hopl et al., 2024). Our previous studies further indicate that proposed structural changes along the TM3/TM4 interface are preceded by a widening of the nexus‐TM3 interface in the immediate vicinity of the STIM1 coupling region (Söllner et al., 2026). The hypothesis that Orai1 activation is accompanied by widening of the TM3/TM4 interface is also supported by previously reported targeted MD simulation studies showing a dilation of peripheral TM interfaces (Guardiani et al., 2021) as an early conformational rearrangement conducive to pore dilation. In summary, the current consensus is that STIM1 binding to—mainly—the Orai1 C‐terminus, induces conformational changes that involve widening of the peripheral TM interfaces, namely first within the nexus‐TM3 (Söllner et al., 2026) interface followed by the TM4‐TM3 interface (Hopl et al., 2024; Najjar et al., 2025). These structural changes contribute to the transmission of the opening signal to the pore and can be interrogated systematically by substitutions with bulky or charged amino acids in the TM3/TM4 interface independent of the presence of STIM1.

In addition to the widening of peripheral TM interfaces, sterics along the TM3/TM4 interface are critical determinants fine‐tuning STIM1‐induced Orai1 pore opening. Most prominently, Phe at position 181 does not allow neither STIM1‐mediated activation nor weak constitutive activity. Based on the homology of human Orai1 derived from the X‐ray structure (PDB: 4HKR), this inhibitory effect is possibly induced by an interplay with F250 in TM4 due to spatial proximity. Indeed, mutational screening revealed that the inhibitory effect of V181F is abrogated upon substitution of F250, but not F253, with smaller, less hydrophobic amino acids. In support, contact frequency analysis of MD simulations of Orai1 and Orai1 V181F revealed that F250 predominantly forms contacts with TM3. In contrast, F253 shows more contacts with TM2, in particular TM2′, and the lipid membrane. A comparison of the MD simulations of Orai1 V181F and Orai1 wt in terms of V181/F181 contacts revealed a shift in contact patterns reducing TM3–TM4 contacts and increasing TM3–TM2′ contacts. Hence, inhibition of V181F might be further supported by its contacts with TM2′. Interestingly, the V181F‐induced inhibition could also be overcome by introducing bulkier amino acids (e.g., W) in the vicinity of V181F (e.g., F253), possibly by steric interplay with TM2′, thus leading to an allosteric rearrangement of V181F and F250. Moreover, UV irradiation of mutants containing a photocrosslinking UAA at V181 enhances currents, which is likely due to steric effects. Although non‐specific reactions such as ring expansion of Azi cannot be fully ruled out (Budyka, 2007; Gritsan & Platz, 2009; Leyva et al., 2022), robust, UV‐mediated effects that are specific to the experimental conditions (position of UAA incorporation, in the absence or presence of STIM1; Najjar et al., 2025; Söllner et al., 2026; Maltan et al., 2023) suggest that the formation of photocrosslinks is rather likely. Overall, the steric inhibition caused by V181F/Azi and F250 can be overcome either by reducing hydrophobicity and size, or separately by further significant increase in the side chain size (e.g., F253W) or other steric effects (Azi).

Beyond the inhibitory impact of sterics, we demonstrate clear evidence for the additional role of hydrophobicity along the TM3/TM4 interface in modulating pore opening in both, Orai1 V181F and Orai1 wt. This is particularly established by an extended hydrophobic core stretching over two helical turns (aa F250–253) along the TM3/TM4b interface, located close to the P245 kink. Indeed, the removal of hydrophobic residues on TM3 (V181, L185) alone (Derler et al., 2018; Fahrner et al., 2018) or independently also in TM4 (F250, F253) alone (Tiffner et al., 2021b), led to weak constitutive activity. Notably, activation levels of triple (V181S F250S F253S) or double serine (L185S F250S) substitutions exceed those of Orai1 wt in the presence of STIM1 (Hopl et al., 2024). The less pronounced enhancement of Orai1 L185S F250S activation levels compared to Orai1 wt in the presence of STIM1, may be due to L185's position deeper within the membrane compared to V181, allowing residual hydrophobic interactions near the cytosolic side to persist in the Orai1 L185S F250S mutant. Strongly enhanced hydrophobicity can be achieved by triple insertion of Ile at V181, F250 and F253. This triple mutant does not exhibit STIM1‐mediated activation. In further support, aliphatic single point mutants (e.g., Orai1 A254L) show reduced STIM1‐mediated activation compared to Orai1 wt, potentially due to a reduced conformational response of Orai1 upon STIM1 coupling. Notably, aromatic mutations at A254 show enhanced STIM1‐mediated activation compared to Orai wt, likely due to the above mentioned sterically motivated dilation effect (Najjar et al., 2025). In accord with these findings, we previously demonstrated that increased hydrophobicity along the nexus‐TM3 interface, in particular the L174‐L261 contact site, abolished STIM1‐mediated Orai1 activation (Söllner et al., 2026). Overall, these findings reveal that hydrophobicity in the TM3/TM4 interface retains Orai1 in the closed but activatable state. In contrast, if hydrophobicity in the TM3/TM4 interface gets too high, STIM1‐induced activation is entirely abolished. However, a decrease in hydrophobicity along the TM3/TM4 interface enhances activatability by STIM1 and can also significantly exceed STIM1‐induced Orai1 wt current levels in special cases.

At a more quantitative level, we find a near perfect correlation between current densities and hydrophobicity for F250 and F253 variants based on the Eisenberg scale (SI). The Eisenberg scale models hydrophobicity based on solvent exposed peptide models and globular proteins (Eisenberg et al., 1984; Roseman, 1988) as well as TM protein complexes (Eisenberg et al., 1984). We hypothesize that the change of the chemical environment of F250 and F253 during Orai1 activation may involve enhanced interactions with nearby polar and charged moieties. For instance, charged phosphates of phospholipid headgroups may be able to more efficiently polarize the environment of F250 and F253 in the active state of Orai1. Moreover, we cannot rule out an enhancement of water interactions around F250 and F253 during STIM1‐induced activation, which has been implicated computationally for Orai1 V181K without STIM1 (Hopl et al., 2024). The putatively enhanced polarity of the F250/F253‐environment may justify the success of the Eisenberg scale, compared to other scales (SI) (Eisenberg et al., 1984) such as UHS/MHS scale, which primarily focus on transmembrane domains in a nonpolar environment.

The observed UV effects may imply that photocrosslinking‐induced activation is a result of reduced hydrophobicity of the reactive Azi group. Indeed, Azi's dipole moment enhances upon excitation from 2.02 to 4.42 Debye (Boča et al., 2023) (see also Tables S1, S2) indicating increased hydrophilicity. However, covalent bond formation, relaxation, and any nonspecific binding events are expected to occur in the range of 1 ns (Aydin & Coin, 2023; Leyva et al., 2022), thus, ~10 orders of magnitude faster than the ensuing Ca^2+^‐dependent functional response (10–15 min). Based on this timing, we conclude that UV‐induced chemical reactions of Azi dominate over local hydrophobicity changes in determining channel behavior. In support, we do not find a correlation between UV‐induced current densities and hydrophobicity, but only side chain size. Hence, we propose that steric constraints and conformational rearrangements imposed by photocrosslinking at the TM3/TM4 interface likely outweigh local transient hydrophobicity changes in governing activation.

In summary, our findings reveal a local subtle balance of physico‐chemical properties along the TM3/TM4 interface required for Orai1 pore opening, namely steric restraints and hydrophobicity. On the one hand, widening of the TM3/TM4 interface is a critical conformational change conducive to pore opening. On the other hand, hydrophobic residues along the TM3/TM4 interface play a many‐faceted role. First, they maintain a de‐wetted interface that stabilizes the closed state of Orai1. Second, their hydrophobic character appears evolutionarily (Ben Chorin et al., 2020) balanced to be permissive enough for STIM1‐mediated activation on the one hand but also to be stable enough to avoid overactivation on the other hand (Najjar et al., 2025). Nonetheless, further research is required to clarify the conformational propagation of the STIM1 activation signal within Orai1.

## 4 MATERIALS AND METHODS

### 4.1 Molecular biology

Human Orai1 (Orai1; accession number NM_032790, kindly provided by the laboratory of A. Rao) constructs were cloned into the pEYFP‐C1 (Clontech) expression vector via KpnI/XbaI (Orai1) restriction sites for C‐terminal fluorescent labeling. QuikChange™ XL site‐directed mutagenesis kit (Stratagene) was used to perform site‐directed mutagenesis of all the mutants, with the corresponding Orai1 constructs serving as a template.

Human STIM1 (STIM1; Accession Number: NM_003156), N‐terminally ECFP‐tagged, was kindly provided by T. Meyer's Lab, Stanford University. The integrity of all resulting clones was confirmed by sequence analysis (Eurofins Genomics/Microsynth).

### 4.2 Cell culture and transfection

Human embryonic kidney 293 (HEK293 [#ACC305]) was purchased from DSMZ (German Collection of Microorganisms and Cell Culture). CRISPR‐Cas9 STIM1/STIM2 double‐knockout (DKO) HEK293 (Najjar et al., 2025) cells were cultured in DMEM as recommended by the DSMZ. The media were supplemented with L‐glutamine (2 mM), streptomycin (100 μg/mL), penicillin (100 units/mL), and 10% fetal calf serum while growing at 37°C in a humidity‐controlled incubator with 5% CO_2_. CRISPR‐Cas9 STIM1/STIM2 DKO HEK293 cells were kindly provided by Mohammed Trebak (Najjar et al., 2025).

Transient transfection was performed (Najjar et al., 2025) using TransFectin Lipid Reagent (Bio‐Rad Laboratories; 2 μL per transfection). The plasmid ratio used in the experiments was 1 μg Orai1:2 μg tRNA/aaRS pair for normal HEK293 cells. In the presence of STIM1, 1 μg STIM1 and for Ca^2+^‐imaging, 1 μg R‐GECO1.2 were used in addition. To circumvent lower expression levels, in STIM1/STIM2 DKO HEK293 cell lines the amount of Orai1 plasmids was raised to 1.5 μg. Experiments were performed 18–24 h after transfection. Throughout the study, we used Orai1‐wild‐type/mutant labeled C‐terminally with YFP and, where applicable, CFP‐STIM1. The growth media of all transfected cells were supplemented with the specific UAA (1 mM; *p*‐azido‐L‐phenylalanine [MedChemExpress] or *p*‐benzoyl‐L‐phenylalanine [BACHEM], dissolved in 0.5 M NaOH). Potential mycoplasma contamination was checked regularly using the VenorGeM Advanced Mycoplasma Detection Kit (VenorGeM).

### 4.3 Ca^2+^ imaging

HEK293 cells were transfected with Orai1/R‐Geco1.2, and grown on coverslips for 1 day. Coverslips were transferred to a cell bath and covered with an extracellular solution without Ca^2+^ and mounted on an Axiovert 135 inverted microscope (Zeiss, Germany) with a mounted sCMOS‐Panda digitale Scientific Grade camera 4.2 MPixel and a LedHUB LED Light‐Engine light source. R‐Geco1.2 was excited using the LED spanning 500–600 nm together with a Chroma filter allowing excitation between 540 and 580 nm and emission between 590 and 660 nm. Results of Ca^2+^ measurements are shown as normalized intensities of R‐Geco1.2 fluorescence in HEK293 cells (Maltan et al., 2023). Image acquisition and intensity recordings were performed with Visiview5.0.0.0 software (Visitron Systems). Store depletion was induced by addition of 1 μM thapsigargin in extracellular solution. A Thomas Wisa perfusion pump was used for extracellular solution exchange during the experiment (Maltan et al., 2023). All experiments were performed on different 3 days at standard laboratory conditions using extracellular solutions containing (in mM): 140 NaCl, 10 HEPES, 10 glucose, 5 KCl, 1 MgCl_2_, pH 7.4 and 0 or 1 CaCl_2_, respectively.

### 4.4 Electrophysiology

Electrophysiological recordings were performed using an inverted microscope (Zeiss Axiovert 200), an Axopatch 200B amplifier (Molecular Devices), a PatchStar micromanipulator (Scientifica), and a Lumencor Spectra III light engine controlled by SPECTRA III 8‐LCR‐VA v1.5.22 (Lumencor). Electrophysiological recordings were acquired with pClamp11 software.

After transient transfection of HEK293 cells, the cells were incubated for 4 h at 36°C, 90% RH, and 5% CO_2_. The cells were then reseeded onto cover slips, and incubated again for 18 h. Translocation, expression patterns, and levels of the various constructs were carefully monitored by fluorescence microscopy and were not significantly changed by the introduced mutations. Electrophysiological experiments were performed at 18–20°C, using the patch‐clamp technique in the whole‐cell recording configuration. For Orai1, STIM1/Orai1 current measurements, voltage ramps were applied every 5 s from a holding potential of 0 mV, covering a range from −90 to +90 mV over 1 s. For the determination of FCDI, voltage steps were applied to −70/−90/−110 mV for 2000 ms starting from a holding potential of 0 mV. To induce passive store depletion, the solution within the pipette contained (in mM) 3.5 MgCl_2_, 145 cesium methane sulfonate, 8 NaCl, 10 HEPES, and 20 EGTA at pH 7.2. The extracellular solution consisted of (in mM) 145 NaCl, 5 CsCl, 1 MgCl_2_, 10 HEPES, 10 glucose, and 10 CaCl_2_ at pH 7.4. Applied voltages were not corrected for the liquid junction potential, which was determined as +12 mV. All currents were leak‐corrected by subtraction of the leak current which remained following 10 μM La^3+^ application. All experiments were carried out at least on three different days. Bar graphs in the figures display maximum current densities for Orai1 proteins in the absence and presence of STIM1.

As regards the UAA mutants, appropriate controls were carried out in our previous studies (Maltan et al., 2023; Söllner et al., 2026), for example with regard to the quantification of expression levels, cell viability following UV irradiation, or control experiments involving UV irradiation alone on wild‐type channels. UV illumination was obtained using the 365 nm LED (Lumencor Spectra III) through the objective (“Fluar” 20×/0.75 M27) with a UV intensity of 2.2 mW/cm^2^ at the cell. A dual bandpass filter (365 + 475 nm) was used within all experiments.

### 4.5 Confocal FRET fluorescence microscopy

Confocal FRET microscopy was carried out at room temperature 18–24 h after transfection. The standard extracellular solution contained (in mM): 145 NaCl, 5 KCl, 10 HEPES, 10 glucose, 1 MgCl_2_, 2 CaCl_2_ and was set to pH 7.4. For Ca^2+^ store depletion, a Ca^2+^‐free extracellular solution containing 1 μM thapsigargin or 10 μM BHQ was used. The experimental setup consisted of a CSU‐X1 Real‐Time Confocal System (Yokogawa Electric Corporation, Japan) combined with two CoolSNAP HQ2 CCD cameras (Photometrics, AZ, USA). The installation was also fitted with a dual port adapter (dichroic, 505lp; cyan emission filter, 470/24; yellow emission filter, 535/30; Chroma Technology Corporation, VT, USA). An Axio Observer.Z1 inverted microscope (Carl Zeiss, Oberkochen, Germany) and two diode lasers (445 and 515 nm, Visitron Systems, Puchheim, Germany) were connected to the described configuration. All described components were positioned on a Vision IsoStation antivibration table (Newport Corporation, CA, USA). A perfusion pump (ASF Thomas Wisa, Wuppertal, Germany) was used for extracellular solution exchange during experiments. Image recording and control of the confocal system were carried out with the VisiView software package (v.2.1.4, Visitron Systems). The illumination times for individual sets of images (CFP, YFP, FRET) that were recorded consecutively with a minimum delay were kept in a range of 100–300 ms. Due to cross‐excitation and spectral bleed‐through, image correction before any FRET calculation was required. YFP cross‐excitation (a) and CFP crosstalk (b) calibration factors were therefore determined on each measurement day using separate samples in which cells only expressed CFP or YFP proteins. FRET analysis was limited to pixels with a CFP:YFP ratio between 0.1:10 and 10:0.1. After this threshold determination as well as background signal subtraction, the apparent FRET efficiency *E*_app_ was calculated on a pixel‐to‐pixel basis. This was performed with a custom program integrated into MATLAB (v.7.11.0, The MathWorks, Inc., MA, USA) according to the following equation:

$$E_{\mathrm{app}}=\frac{I_{\text{FRET}}-aI_{\mathrm{YFP}}-bI_{\mathrm{CFP}}}{I_{\text{FRET}}-{\mathrm{aI}}_{\mathrm{YFP}}+\left(G-b\right)I_{\mathrm{CFP}}}$$

where *I*_FRET_, *I*_YFP_, and *I*_CFP_ denote the intensities of the FRET, YFP, and CFP images, respectively. *G* denotes a microscope‐specific constant parameter that was experimentally determined as 2.75.

### 4.6 NFAT‐translocation studies using confocal fluorescence microscopy

Images of Orai1 and Orai1‐mutants as well as CFP‐NFAT (NFATc1; Kar et al., 2011) localization were created and analyzed with a custom‐made software integrated into MATLAB (v7.11.0, The MathWorks, Inc., MA, USA). ImageJ (Schneider et al., 2012) was employed for subcellular localization analysis of the NFAT transcription factors by intensity measurements of the cytosol and nucleus, distinguishing between three different populations with different nucleus/cytosol ratios: inactive (<0.85), homogenous (0.85–1.15), and active (>1.15) (Schober et al., 2019).

NFAT measurements were performed 18–24 h after cell transfection and conducted in media containing 2 mM Ca^2+^. For time‐dependent NFAT translocation, cells were transfected and grown in 0 mM Ca^2+^ medium for 18–24 h. After this time the media was exchanged by 2 mM extracellular Ca^2+^ solution and incubated for 2 h. For steady state experiments, cells were incubated in 2 mM Ca^2+^ containing media for 18–24 h. Media was replaced with 2 mM Ca^2+^ solution before measurement.

### 4.7 Molecular dynamics simulations

Our molecular dynamics simulations employed a homology model of the Orai1 channel (Frischauf et al., 2017) that is based on the crystal structure of Orai1 in *D. melanogaster* (Hou et al., 2012). We embedded the channel in a membrane composed of lipids DDPC, DLPE, DLPG, DLPS, DMPI, and PSM at ratio 40:18:7:20:10:15 (Dawaliby et al., 2016). The system was solvated in TIP3P water in a simulation box with spanning 156 × 156 × 121 Å^3^. We added sodium and chloride ions at a concentration of 150 mM as well as 2 mM CaCl_2_. The system was equilibrated using the CHARMM‐GUI seven‐step procedure (Jo et al., 2008), with a 50‐ns conventional NpT equilibration. Simulations were performed at 300 K using NAMD 2.14 (Phillips et al., 2005) and the CHARMM36m force field (Huang et al., 2016). The temperature was regulated using Langevin dynamics with a damping coefficient of 1/ps. The pressure was set to 1 atm with the Langevin piston method, using an oscillation period of 100 fs and damping timescale of 50 fs. Long‐range electrostatic interactions were handled with the particle‐mesh Ewald method (Essmann et al., 1995). The V181F mutation was implemented using CHARMM (Brooks et al., 2009). After equilibration, we performed four independent simulation replicas à 300 ns for both the wt and the V181F mutant.

For our inter‐domain contact analysis, we quantified how frequently a given residue *r* interacts with each transmembrane domain *D*. For each trajectory frame, we computed inter‐residue contacts based on the minimum heavy‐atom distance between residue pairs, using a cutoff of 4.5 Å. Contacts involving residues within five sequence positions of *r* were excluded to avoid trivial local contacts. For every frame, we identified all partner residues that form contacts with *r* and assigned each partner to its corresponding transmembrane domain or to the membrane. We then calculated, at each frame, the fraction of contacts that involved partner residues belonging to domain *D*. Contact fractions were collected for all six Orai1 subunits and concatenated. Figure 3m displays the mean and standard deviation of the contact fractions across the simulation time and across all subunits. Contact maps were calculated with MDTraj (McGibbon et al., 2015).

### 4.8 Hydrophobicity profiles

Hydrophobicity profiles were determined based on Roseman (Roseman, 1988), Kyte and Doolittle (Kyte & Doolittle, 1982), Eisenberg (Eisenberg et al., 1984), and UHS and MHS (Koehler et al., 2009).

### 4.9 Statistics and reproducibility

Results are presented as mean value ± SEM calculated for the indicated number *n* of experiments. For statistical comparison, the Mann–Whitney test was performed for comparison of two independent samples considering differences as statistically significant at *p* < 0.05. Levene test was used to test for variance homogeneity. If fulfilled, one‐way ANOVA test was used for statistical comparison of multiple independent samples using the F‐distribution. If not fulfilled, the Welch‐ANOVA test was used instead. Subsequently, Fisher's least significant post‐hoc test was used after one‐way ANOVA, while Games‐Howell post hoc test was used after Welch‐ANOVA to determine the pairs that differ statistically significant (*p* < 0.05). In all cases, the Shapiro–Wilk test was applied to prove normal distribution of the respective datasets (or one‐sample Kolmogorov–Smirnov test). F and *p* values of statistics performed are included in Table S6 for all samples.

All Ca^2+^ imaging experiments were performed on 3 days in paired comparison leading to similar results. All electrophysiological experiments were carried out on at least two different days in paired comparison leading to similar results. Cell images were taken for each experiment showing comparable cellular distribution of the respective proteins/mutants, as shown in one representative image in the respective figures.

## AUTHOR CONTRIBUTIONS

Maximilian Fröhlich, and Isabella Derler conceived and coordinated the study and wrote the paper. Maximilian Fröhlich, Tamara Radišković, Valentina Hopl, Hadil Najjar, Diana Thallinger, and Adéla Tiffner performed and analyzed electrophysiological experiments. Selina Harant performed and analyzed Ca^2+^ imaging experiments. Magdalena Prantl, Herwig Grabmayr, and Matthias Sallinger carried out fluorescence microscopy experiments. Muhammad Shehzad Anwar, and Magdalena Prantl conducted NFAT translocation experiments. Maximilian Fröhlich, Valentina Hopl, Julia Söllner, Yuliia Nazarenko, Selina Harant, and Diana Thallinger contributed to molecular biology and biochemistry. Ferdinand Horvath, and Heinrich Krobath performed and supervised MD simulations. All authors reviewed the results and approved the final version of the manuscript.

## Supporting information


**Figure S1.** Function of various single and double mutants containing substitutions around V181, I182 and A254.
**Figure S2.** STIM1‐ und UV‐induced activation of Orai1 V181Azi/Bpa triple mutants.
**Figure S3.** STIM1‐induced activation properties of single, double and triple substitutions around V181.

**Table S1.** Electrostatic moments of the Azi amino acid in the ground state after density functional theory optimization of atomic coordinates using B3LYP and aug‐cc‐pVDZ in implicit water. The leading term is a dipole term with a net dipole moment of 2.02 Debye.
**Table S2.** Partial charge fit result of the neutral optimized Azi singlet excited state. The xyz columns denote the optimized coordinates in angstrom. The right most column shows the atomistic charge modeled at the very atom side. Below the table we summarize the net dipole moment of the Azi variant based on the DFT calculation (exact) and the partial charge fit. The nitrene neutrogen partial charge (−0,66 e) is highlighted with the triple exclamation mark.
**Tables S3–S5.** Summary of all calculated Pearson correlation coefficients. Table S3: Cross correlation between all hydrophobicity scales and VVdW used for correlation analysis with current densities. Tables S4–S5: Correlation between current densities of experimentally investigated Orai1 triple mutant subsets and all hydrophobicity scales or VVdW. Tabular overview of correlation plots yielding correlation coefficients summarized in Tables S3–S5. The maximum normalized inward current density has been correlated against different hydrophobicity scales and VVdW volume (lowest line). The hydrophobicity scales are dimensionless due to normalization. Data points are average values based on repeated experiments of *n* = 7–15 (Figure 4). Error bars represent SEM. The vertical dashed lines are guides for the eye. The dashed dotted colored line is a linear fit of the data points. In the respective plots we represent the Pearson correlation coefficient R, which are all summarized in Tables S3–S5.

**Table S6.** Table provides F and p values of statistics performed for the data sets in the respective main and supplementary figures.

## Data Availability

The data that support the findings of this study will be openly available in zenodo repository as soon as the manuscript is accepted.
