# Supplementary material for: Evolutionarily conserved hydrophobicity and sterics in TM3/TM4 balance Orai1 pore opening
Source: Protein Sci. 2026 Jul 12;35(8):e70684. doi: 10.1002/pro.70684 (PMC13358370; doi:10.1002/pro.70684)
Supplement: Supplementary file 1 — Figure S1. Function of various single and double mutants containing substitutions around V181, I182 and A254. Figure S2. STIM1‐ und UV‐induced activation of Orai1 V181Azi/Bpa triple mutants. Figure S3. STIM1‐induced activation properties of single, double and triple substitutions around V181. [file PRO-35-e70684-s003.pdf]

Supplementary Figures

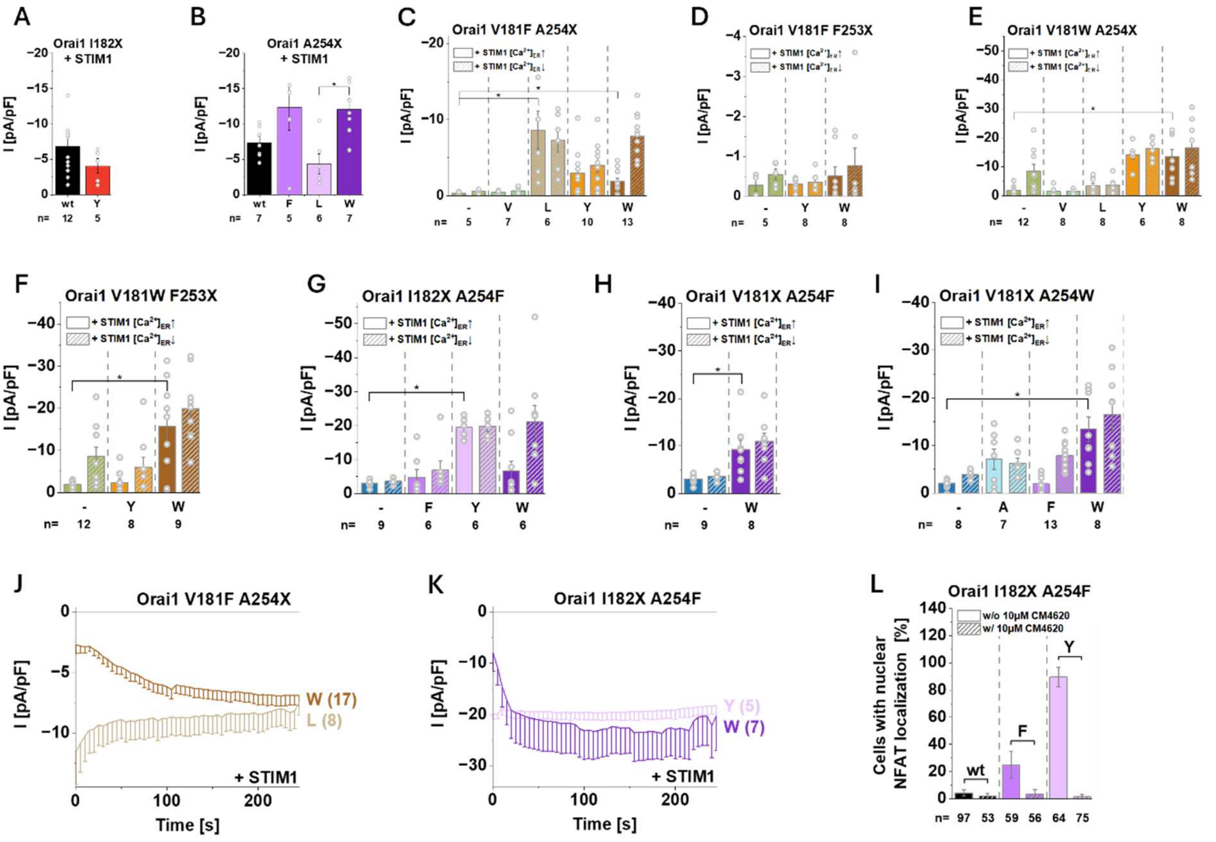

Suppl. Figure 1: Function of various single and double mutants containing substitutions around V181, I182 and A254. A, B Bar graphs showing maximum current densities of Orai1 I182Y (**A**) and Orai1 A254F/L/W (**B**) compared to Orai1 wt in the presence of STIM1. **C – I** Bar graphs showing maximum current densities before and after STIM1 binding of the Orai1 V181F A254V/L/Y/W (**C**), Orai1 V181F F253Y/W (**D**), Orai1 V181W A254V/L/Y/W (**E**), Orai1 V181W F253Y/W (**F**), Orai1 I182F/Y/W A254F (**G**), Orai1 V181W A254F (**H**), and Orai1 V181A/F/W A254W (**I**) compared to the respective single mutant. **J, K** Time courses of current densities of Orai1 V181F A254W/L (**J**) and Orai1 I182Y/W A254F (**K**) in the presence of STIM1. **L** Bar graphs showing the percentage of cells with nuclear NFAT translocation for the Orai1 I182F/Y A254F with and without 24h incubation with CM-4620 compared to Orai1 wt.

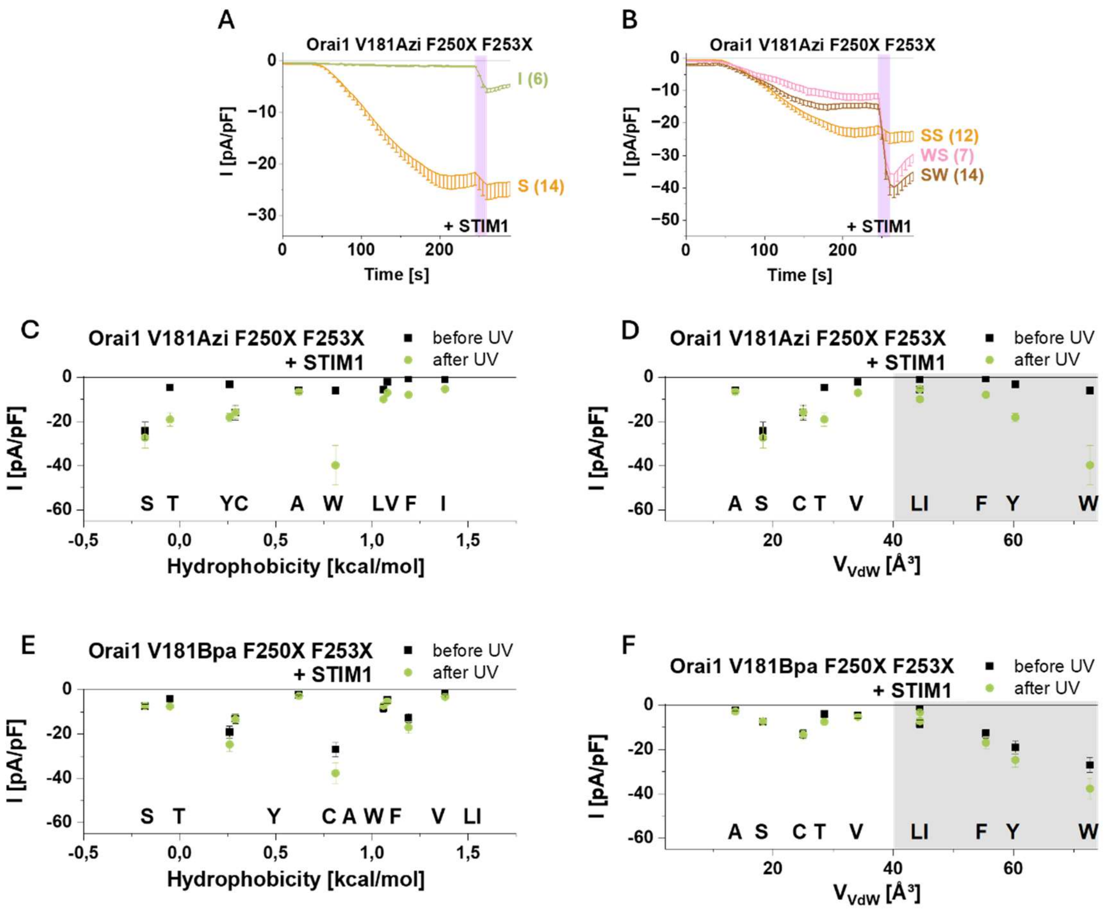

Suppl. Figure 2: STIM1- und UV-induced activation of Orai1 V181Azi/Bpa triple mutants. A, B Time courses of current densities of Orai1 V181Azi F250I/S F253I/S (**A**) and Orai1 V181Azi F250S/S/W F253S/W/S (**B**) in the presence of STIM1. The purple bar represents the illumination with UV-light (365nm) for 15s. **C – F** Dot blots showing maximum current densities of Orai1 V181Azi/Bpa F250X F253X (X = A, S, C, T, V, L; I, F, Y, and W) in the presence of STIM1 before (black dots) and after (green dots) 15s UV-light (365nm) exposure. Current densities were plotted over hydrophobicity (**C** and **E**) and Van-der-Waals volume (**D** and **F**) of the substituted residue.

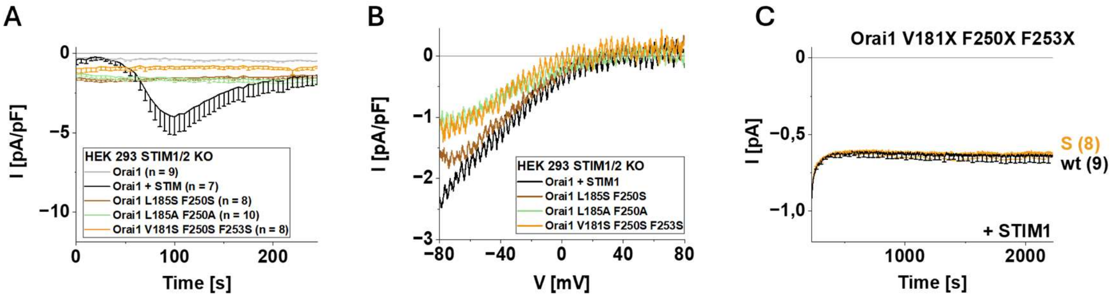

Suppl. Figure 3: STIM1-induced activation properties of single, double and triple substitutions around V181. A Time course of current densities of Orai1 L185A/S F250A/S and Orai1 V181S F250S F253S in absence of STIM1 compared to Orai1 wt in the presence and absence of STIM1, measured in HEK293 STIM1/STIM1 double knock out (KO) cells. **B** I/V relationship of current densities of Orai1 L185A/S F250A/S and Orai1 V181S F250S F253S in absence of STIM1 compared to Orai1 wt in presence of STIM1, measured in HEK293 STIM1/STIM2 double KO cells. Inlet represents reversal potential (V_{rev}) of wild-type STIM1 + Orai1 currents versus Orai1 L185S/A F250S/A and Orai1 V181S F250S F253S. **C** Time course of currents of the Orai1 mutant V181S F250S F253S compared to Orai1 wt showing fast Ca^{2+} dependent inactivation (FCDI) in the presence of STIM1.
