# Supplementary material for: Evolutionarily conserved hydrophobicity and sterics in TM3/TM4 balance Orai1 pore opening
Source: Protein Sci. 2026 Jul 12;35(8):e70684. doi: 10.1002/pro.70684 (PMC13358370; doi:10.1002/pro.70684)
Supplement: Supplementary file 2 — Table S1. Electrostatic moments of the Azi amino acid in the ground state after density functional theory optimization of atomic coordinates using B3LYP and aug‐cc‐pVDZ in implicit water. The leading term is a dipole term with a net dipole moment of 2.02 Debye. Table S2. Partial charge fit result of the neutral optimized Azi singlet excited state. The xyz columns denote the optimized coordinates in angstrom. The right most column shows the atomistic charge modeled at the very atom side. Below the table we summarize the net dipole moment of the Azi variant based on the DFT calculation (exact) and the partial charge fit. The nitrene neutrogen partial charge (−0,66 e) is highlighted with the triple exclamation mark. Tables S3–S5. Summary of all calculated Pearson correlation coefficients. Table S3: Cross correlation between all hydrophobicity scales and VVdW used for correlation analysis with current densities. Tables S4–S5: Correlation between current densities of experimentally investigated Orai1 triple mutant subsets and all hydrophobicity scales or VVdW. Tabular overview of correlation plots yielding correlation coefficients summarized in Tables S3–S5. The maximum normalized inward current density has been correlated against different hydrophobicity scales and VVdW volume (lowest line). The hydrophobicity scales are dimensionless due to normalization. Data points are average values based on repeated experiments of n = 7–15 (Figure 4). Error bars represent SEM. The vertical dashed lines are guides for the eye. The dashed dotted colored line is a linear fit of the data points. In the respective plots we represent the Pearson correlation coefficient R, which are all summarized in Tables S3–S5. [file PRO-35-e70684-s001.pdf]

Supplementary Information

Density functional theory calculations

We performed now density functional theory calculations at the B3LYP level[1–3] with the basis set aug-cc-pVDZ[4,5] on the ground state Azi and the photoactivated Azi amino acid in implicit water (ε_r = 80) using the integral equation formalism of the polarizing continuum model (IEF-PCM). We extracted the net dipole moments of the optimized neutral singlet ground state (SI Table 1) and the singlet photoactivated state of Azi (with capped backbone i.e. acetylated amino moiety, N-methyl-amidated carboxy moiety; SI Table 2). Moreover, we performed partial charge fitting using the CHELP-BOW methods[6] to assess local polarization around the nitrene group in the photoactivated state (SI Table 2). The Azi ground state partial charges can be readily achieved from Smith et al.[7.]

SI Table 1: Electrostatic moments of the Azi amino acid in the ground state after density functional theory optimization of atomic coordinates using B3LYP and aug-cc-pVDZ in implicit water. The leading term is a dipole term with a net dipole moment of 2,02 Debye.

```
 ----------------------------------------------------------------
                 Cartesian Multipole Moments
 ----------------------------------------------------------------
    Charge (ESU x 10^10)
                 -0.0000
    Dipole Moment (Debye)
         X      -1.2593      Y      -1.5844      Z       0.0136
       Tot       2.0239
    Quadrupole Moments (Debye-Ang)
        XX    -126.2545     XY       7.7568     YY    -111.2974
        XZ      -6.6494     YZ      14.6169     ZZ    -115.1049
    Octopole Moments (Debye-Ang^2)
       XXX     -85.8377    XXY      16.0372    XYY       7.5183
       YYY     -37.6054    XXZ      56.9541    XYZ     -52.1789
       YYZ     -15.3799    XZZ      21.0519    YZZ      13.2411
       ZZZ     -46.1404
    Hexadecapole Moments (Debye-Ang^3)
      XXXX   -7330.5452   XXXY     821.6050   XXYY   -1505.0255
      XYYY     692.8339   YYYY   -1231.6228   XXXZ     210.7907
      XXYZ     152.4767   XYYZ     126.6681   YYYZ       6.0483
      XXZZ   -1307.0647   XYZZ     172.6812   YYZZ    -391.2360
      XZZZ     508.7121   YZZZ      -7.8451   ZZZZ    -957.5298
 ----------------------------------------------------------------
```

SI Table 2: Partial charge fit result of the neutral optimized Azi singlet excited state. The xyz columns denote the optimized coordinates in angstrom. The right most column shows the atomistic charge modelled at the very atom side. Below the table we summarize the net dipole moment of the Azi variant based on the DFT calculation (exact) and the partial charge fit. The nitrene neutrogen partial charge (-0,66 e) is highlighted with the triple exclamation mark.

#	atom	x	y	z (au)	Fitted	
1	N	2.371009	-1.382585	0.375952	-0.515220	
2	C	1.243780	-0.568720	-0.027880	-0.112083	
3	C	1.597932	0.889710	0.290733	0.584906	
4	O	2.300039	1.167069	1.263228	-0.521544	
5	N	1.079422	1.824101	-0.541189	-0.301165	
6	C	-0.057028	-1.004661	0.725831	-0.238122	
7	H	1.086481	-0.713617	-1.103807	0.086909	
8	C	-1.307256	-0.368492	0.185419	-0.070137	
9	H	0.069878	-0.771703	1.790498	0.139322	
10	H	-0.117556	-2.094258	0.609653	0.139322	
11	C	-1.982891	0.637318	0.911034	0.061526	
12	C	-3.166943	1.172776	0.436139	-0.495853	
13	C	-1.808722	-0.773714	-1.080366	0.080761	
14	C	-2.877011	-0.120485	-1.657234	-0.537361	
15	C	-3.632910	0.879791	-0.912509	0.941044	
16	H	-1.597424	0.935225	1.887621	0.112948	
17	H	-3.745482	1.894207	1.012449	0.194374	
18	H	-1.297215	-1.573451	-1.619365	0.143322	
19	H	-3.233129	-0.374592	-2.655775	0.204920	
20	N	-4.644077	1.523053	-1.415912	-0.661427	!!!
21	C	2.578533	-2.628669	-0.139710	0.845080	
22	H	2.984671	-0.979033	1.073378	0.294762	
23	O	1.813950	-3.119068	-0.969945	-0.596874	
24	C	3.803305	-3.364767	0.365049	-0.765035	
25	H	4.458133	-3.580071	-0.489174	0.205344	
26	H	3.485211	-4.325752	0.788532	0.205344	
27	H	4.367216	-2.803446	1.120247	0.205344	
28	C	1.245027	3.252684	-0.303686	-0.349361	
29	H	0.544042	3.619206	0.460045	0.150143	
30	H	1.068751	3.790970	-1.241000	0.150143	
31	H	2.266071	3.444869	0.042434	0.150143	
32	H	0.486060	1.522002	-1.300300	0.268524	
Total charge:					0.000000	-0.000000

Dipole moment (Debye):				
Fitted-od:	4.124538	0.111101	1.567639	4.413802
Fitted:	1.622706	0.043710	0.616752	1.736511
Exact:	4.132700	0.082900	1.565300	4.419983

Correlation Analysis

To check for a potential correlation between our experimentally determined current densities and hydrophobicity or Van der Waals volume (V_{VdW}), we initially chose to compare five different scales to assess their respective efficiency for Orai1 activation measured by current density: the Eisenberg, the Roseman, the Kyte & Doolitte (K&D), the UHS and the MHS scale. The Eisenberg scale relies on a substantial subset of solvated globular proteins as well as transmembrane proteins.[8–11] The Roseman scale mainly focuses on solubility of model peptide at interfaces of water and different solvent systems.[10] K&D scale primarily addresses burial of amino acids in globular proteins.[9] Both UHS and MHS were developed mainly for computational prediction of transmembrane segments, with MHS derived from a limited dataset of 16 mammalian membrane proteins.[11]

In addition, we consider the side chain Van der Waals volume (V_{VdW}) as another potential variable for our correlation analysis. The values were retrieved from Makhatadze et al[12].

In a first step we investigated the correlation between our selected scales, in particular the correlation between V_{VdW} and Eisenberg scale with different amino acid subsets used in our experiments. For the full scales we found a correlation between V_{VdW} and (i) Roseman (-0,43), (ii) Eisenberg (-0,49) and (iii) between Roseman and Eisenberg (0,97; SI Table 3). Hence, V_{VdW} and the hydrophobicity scales are largely uncorrelated, while the Roseman and Eisenberg scale are highly correlated. For a reduced subset of amino acids (A/C/F/I/L/S/T/V/W/Y) we find a correlation between V_{VdW} and (i) Roseman (-0,78), (ii) Eisenberg (-0,43) and (iii) between Roseman and Eisenberg (0,85; SI Table 3). We note that the correlation between the V_{VdW} and Roseman scale is solid, while correlation between V_{VdW} and Eisenberg scale remains uncorrelated. Interestingly, other hydrophobicity scales reveal an even weaker correlation with V_{VdW} scale (SI Table 3), however, K&D, UHS and MHS are based on reduced data sets and specialized settings[9,11] casting doubts on their universal applicability compared to the Eisenberg scale. In summary, we find that the combination of V_{VdW} and Eisenberg scales provides us with two reliable uncorrelated scales for analysis across our amino acid selections (SI Table 3). Note that we do not distinguish between positive and negative correlation, as we are considering the absolute value of the Pearson correlation coefficient.

Next, we correlated hydrophobicity with maximal store-operated and UV-induced (where applicable) current densities of Orai1 V181F/Azi triple mutants using the different hydrophobicity scales discussed above. Taking the larger selection (A/C/F/I/L/S/T/V/W/Y), the Eisenberg and Roseman scales led to weak correlation (~0,7) for the Orai1 V181Azi triple mutant currents (SI Table 4). In contrast, for other scales we found no correlation (~ -0,5 – 0,4; SI Table 4). The smaller amino acid selection (S/C/A/W/V/I) led to near-perfect correlation between hydrophobicity and current densities before UV light exposure in F and Azi triple mutants using Eisenberg and Roseman scale (0,88-0,99), but not other scales (SI Table 4). After UV light, this correlation broke down reliably in all cases (0,05-0,5; SI Table 4). Due to subtle hydrophobicity (re-) weighting in other hydrophobicity scales we find several cases with robust correlation (e.g. K&D after UV) for all selections; this reweighting is reflected by the correlation between K&D and Eisenberg scale of 0,78 (SI Table 3). Similar observations were made for UHS and MHS scales.

For our Bpa variants, we did not find correlations between hydrophobicity and current densities across all mutant subsets (-0,48 – 0,59; SI Table 4). Interestingly, the UHS scales reported weak correlations for the absolute value of current density using the smaller amino acid selection (S/C/A/W/V/I; SI Table 4). Note that our inward currents are defined as negative real numbers formally leading to anti-correlations in our analysis, however, we only consider the absolute Pearson correlation value.

Moreover, we examined the correlation between V_{VdW} and current densities. Most importantly, we found a clear correlation for Azi mutants after UV-light exposure and for the Bpa mutants before and after UV stimulus (-0.7 to -0.86, SI Table 4 & 5). Separately, we also investigated the correlation between V_{VdW} and current densities with photoactive mutants based on a restricted selection of five amino acids (I/L/F/Y/W) because we saw a distinct change in correlation trends in our experimental data starting at the threshold V_{VdW} of Leu (Figure 5, grey shaded area; SI Table 5). With this subset, we could achieve a near perfect correlation (0.99) between V_{VdW} and the absolute value of the inward current. The dataset below the threshold volume yielded a correlation between 0,11 (before UV) and 0,22 (after UV; SI Table 5).

In summary, we consider the Eisenberg scale due to its established universal applicability and its explicit consideration of transmembrane protein complexes[8] as most suitable. In support, we find the Eisenberg and V_{VdW} scales as the best suitable uncorrelated scales for our analysis. We report a strong correlation between hydrophobicity and store-operated current densities for Orai1 V181F/Azi triple mutants before UV light application unlike Bpa mutants. In contrast, corresponding Bpa mutants reveal a stronger correlation between V_{VdW} beyond the threshold volume of Leu and current densities. The dominant effect of size on current densities is in particular pronounced after UV exposure.

SI Table 3 – 5: Summary of all calculated Pearson correlation coefficients. SI Table 3: Cross-correlation between all hydrophobicity scales and V_{VdW} used for correlation analysis with current densities. SI Table 4 – 5: Correlation between current densities of experimentally investigated Orai1 triple mutant subsets and all hydrophobicity scales or V_{VdW}.

SI Table 3

All 20 proteinogenic AA	VdW Volume	Roseman	K&D	Eisenberg	UHS (TM)	MHS (TM)
VdW Volume	1,00					
Roseman	-0,43	1,00				
K&D	-0,43	0,93	1,00			
Eisenberg	-0,50	0,97	0,92	1,00		
UHS (TM)	0,38	-0,98	-0,92	-0,97	1,00	
MHS (TM)	0,41	-0,99	-0,92	-0,97	0,99	1,00

SI Table 4

X = A/C/F/I/L/S/T/V/W/Y	VdW Volume	Roseman	K&D	Eisenberg	UHS (TM)	MHS (TM)
VdW Volume	1,00					
Roseman	0,78	1,00				
K&D	-0,14	0,44	1,00			
Eisenberg	0,43	0,85	0,78	1,00		
UHS (TM)	0,04	-0,47	-0,87	-0,72	1,00	
MHS (TM)	0,11	-0,41	-0,91	-0,79	0,80	1,00

SI Table 5

V181F/Azi/Bpa F250X F253X + S1	X	UV	Roseman	K&D	Eisenberg	UHS (TM)	MHS (TM)	VdW Volume
Azi	A/C/F/I/L/S/T/V/W/Y	before UV	0,69	0,36	0,69	-0,54	-0,51	0,52
Azi		after UV	0,20	0,78	0,50	-0,93	-0,75	-0,34
Bpa		before UV	-0,41	0,51	0,07	-0,53	-0,56	-0,86
Bpa		after UV	-0,41	0,55	0,05	-0,55	-0,56	-0,86
F	S/C/A/W/V/I	-	0,95	0,52	0,99	-0,46	-0,67	0,67
Azi		before UV	0,88	0,53	0,96	-0,49	-0,77	0,49
Azi		after UV	-0,05	0,87	0,35	-0,93	-0,86	-0,58
Bpa		before UV	-0,37	0,62	0,08	-0,79	-0,63	-0,74
Bpa		after UV	-0,49	0,59	-0,05	-0,74	-0,55	-0,82

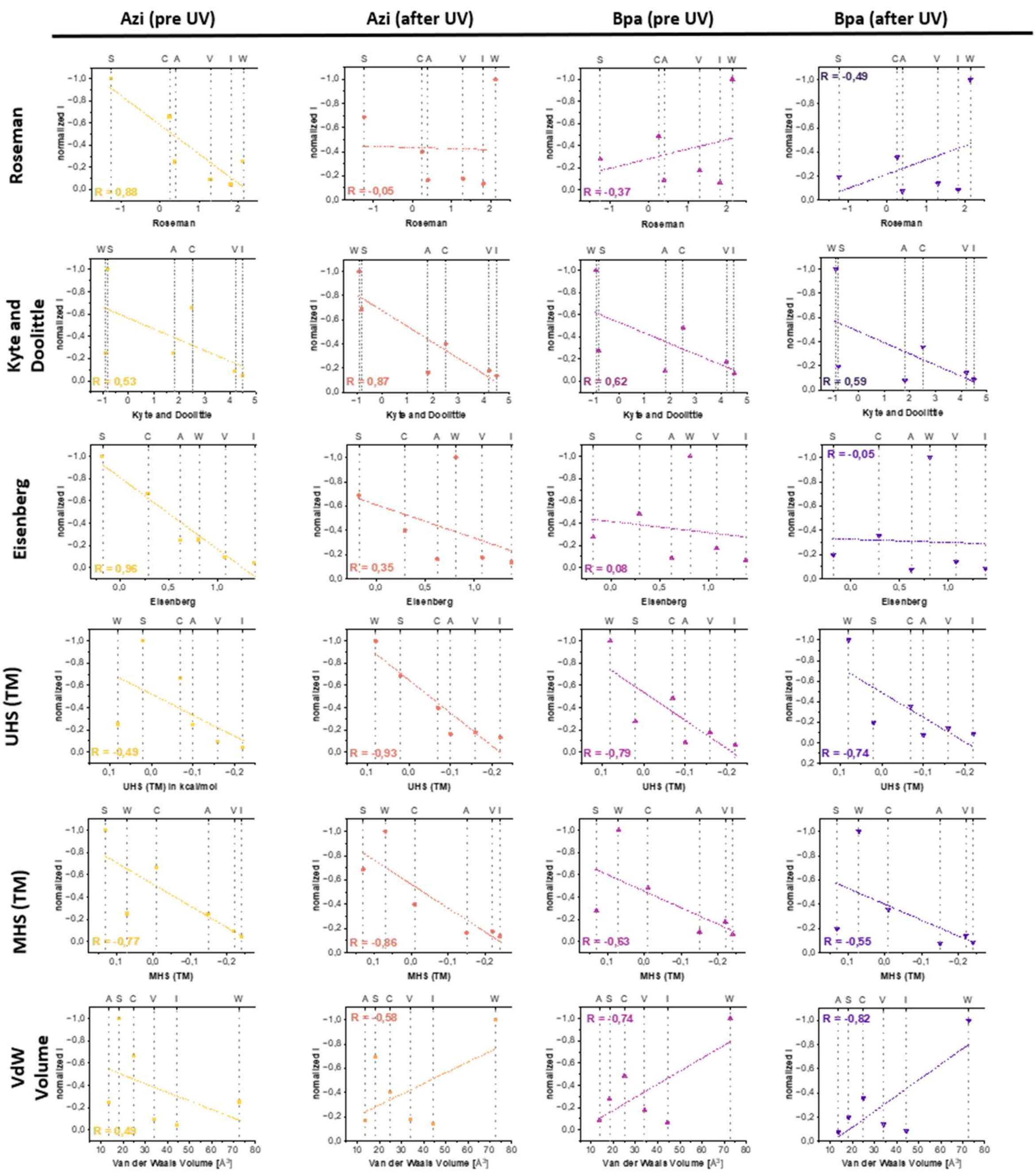

SI Figure 1

Tabular overview of correlation plots yielding correlation coefficients summarized in SI Table 1 – 3. The maximum normalized inward current density has been correlated against different hydrophobicity scales and V_{VdW} volume (lowest line). The hydrophobicity scales are dimensionless due to normalization. Data points are average values based on repeated experiments of n = 7-15 (Fig 4). Error bars represent SEM. The vertical dashed lines are guides for the eye. The dashed dotted coloured line is a linear fit of the data points. In the respective plots we represent the Pearson correlation coefficient R, which are all summarized in the tables 1-3.

References

1. Cramer CJ. Essentials of computational chemistry : theories and models. Wiley; 2014.

2. Stephens PJ, Devlin FJ, Chabalowski CF, Frisch MJ (1994) Ab Initio Calculation of Vibrational Absorption and Circular Dichroism Spectra Using Density Functional Force Fields. J. Phys. Chem. 98:11623–11627.

3. Kim K, Jordan KD (1994) Comparison of Density Functional and MP2 Calculations on the Water Monomer and Dimer. J. Phys. Chem. 98:10089–10094.

4. Tomasi J, Mennucci B, Cancès E (1999) The IEF version of the PCM solvation method: an overview of a new method addressed to study molecular solutes at the QM ab initio level. Journal of Molecular Structure: THEOCHEM 464:211–226.

5. Kendall RA, Dunning TH, Harrison RJ (1992) Electron affinities of the first-row atoms revisited. Systematic basis sets and wave functions. J. Chem. Phys. 96:6796–6806.

6. Sigfridsson E, Ryde U (1998) Comparison of methods for deriving atomic charges from the electrostatic potential and moments. J. Comput. Chem. 19:377–395.

7. Smith AK, Wilkerson JW, Knotts TA (2020) Parameterization of Unnatural Amino Acids with Azido and Alkynyl R-Groups for Use in Molecular Simulations. J. Phys. Chem. A 124:6246–6253.

8. Eisenberg D, Schwarz E, Komaromy M, Wall R (1984) Analysis of membrane and surface protein sequences with the hydrophobic moment plot. J. Mol. Biol. [Internet] 179:125–142. Available from: https://www.sciencedirect.com/science/article/abs/pii/0022283684903097?via%3Dihub

9. Kyte J, Doolittle RF (1982) A simple method for displaying the hydropathic character of a protein. J. Mol. Biol. [Internet] 157:105–132. Available from: https://www.sciencedirect.com/science/article/abs/pii/0022283682905150?via%3Dihub

10. Roseman MA (1988) Hydrophilicity of polar amino acid side-chains is markedly reduced by flanking peptide bonds. J. Mol. Biol. [Internet] 200:513–522. Available from: https://www.sciencedirect.com/science/article/abs/pii/0022283688905402?via%3Dihub

11. Koehler J, Woetzel N, Staritzbichler R, Sanders CR, Meiler J (2009) A Unified Hydrophobicity Scale for Multi-Span Membrane Proteins. Proteins [Internet] 76:13. Available from: https://pmc.ncbi.nlm.nih.gov/articles/PMC2761718/

12. Makhatadze GI, Medvedkin VN, Privalov PL (1990) Partial molar volumes of polypeptides and their constituent groups in aqueous solution over a broad temperature range. Biopolymers 30:1001–1010.
